# Variability of the optical signatures of dissolved organic matter in soils of different mangrove stands (Ouvéa, New Caledonia)

**DOI:** 10.1007/s11356-025-36373-9

**Published:** 2025-04-23

**Authors:** Naïna Mouras, Hugues Lemonnier, Thomas Crossay, Kapeliele Gututauava, Maximilien Mathian, Sarah Louise Robin, Océane Tardivel, Cyril Marchand

**Affiliations:** 1 Institute of Applied and Exact Sciences (ISEA EA7484), University of New Caledonia, 145 Avenue James Cook, Nouville, Nouméa Cedex, BP R4 98851 New Caledonia; 2 Ifremer, UMR 9220, ENTROPIE (IRD, Univ. Réunion, Ifremer, Univ. Nouvelle-Calédonie, CNRS), Nouméa, New Caledonia France; 3 MARBEC, Univ. Montpellier, CNRS, Ifremer, IRD, Sète, France

**Keywords:** Mangrove, Porewater, Carbon cycle, CDOM, Photochemistry, Fluorescence spectroscopy

## Abstract

Mangrove ecosystems are known to play a key role in the global carbon cycle, due to their productivity and their ability for carbon sequestration both in the biomass and in the soil. In the latter, various geochemical processes lead to the production of dissolved organic matter (DOM) that can be exported through tidal pumping and then constitute an important source of organic carbon for adjacent ecosystems. DOM characteristics, and their variabilities, within mangrove soils depend on several factors, including the mangrove species, yet these variations and their origin still need to be precisely constrained. This study examined DOM sources in soils of a carbonate atoll mangrove (Ouvéa, New Caledonia), focusing on two tree species, *Rhizophora apiculata* and *Bruguiera gymnorhiza*, at different growth stages. We analysed porewater properties and DOM optical characteristics through spectroscopic and EEM-PARAFAC methods. Our results indicate distinct TOC and DOC concentrations across species, with mature *B. gymnorhiza* soils showing the highest TOC content (~ 30%) but the lowest DOC content (32 mg L^−1^). These differences seem not to be directly related to site physicochemical conditions (redox, pH, salinity) but may rather reflect differences in DOM sources and production, notably due to different symbiotic relationships with mycorrhizal fungi, which influence microbial activity and organic matter diagenesis. DOM absorbance patterns also varied significantly between species: Beneath *R. apiculata*, DOM had higher protein-like and fulvic-like fluorescence, indicating fresher organic matter, while beneath *B. gymnorhiza*, especially in mature stands, DOM was more humified, suggesting older OM because of a possible long-term accumulation due to the basin-like morphology of this site.

**Supplementary Information:**

The online version contains supplementary material available at 10.1007/s11356-025-36373-9.

## Introduction

Tropical coastal ecosystems play a crucial role in the global carbon cycle by sequestering significant amounts of organic carbon in their soils and through their biogeochemical reactivity (Alongi 2014). Among them, mangroves are key ecosystems, acting both as a sink of atmospheric CO_2_ and a source of organic and inorganic carbon in various forms for adjacent ecosystems (Donato et al. 2011; Kristensen et al. 2008).

Mangroves are widespread, covering an area of around 138,000 km^2^, 75% of which is found along tropical and subtropical coasts (Giri et al. 2011). Globally, these ecosystems are recognized for their exceptional productivity in terms of organic matter (OM) production, estimated at 30.0 Tmol C year^−1^ (Bouillon et al. 2008a; Donato et al. 2011; Kristensen et al. 2008). Comprising around 70 mangrove species (Giri et al. 2011), these ecosystems are characterized by a rich biodiversity, where tree production stands out as the main source of autochthonous organic matter within mangrove forests (Bouillon, et al. 2008b; Kristensen et al. 2008). Accordingly, the organic matter characteristics could display slight variations from one mangrove to another.

Nevertheless, the organic matter of mangrove soils is composed of a variety of organic compounds derived from both autochthonous and allochthonous sources (Alongi 2014; Bouillon, et al. 2008b; Furukawa et al. 1997; Jaffé et al. 2004; Marchand et al. 2016). Allochthonous sources include contributions from both terrestrial and oceanic environments. Microorganisms and fungi are pivotal in decomposing refractory OM, influencing its quality in soils as well as the biogeochemical processes affecting it (Meziane et al. 2006; Zhou et al. 2019).

The biochemistry of mangrove soils has been widely studied with a focus on redox processes in relation to organic content, which are linked to forest type and age (Alongi et al. 1998, 1999; Clark et al. 1998; Marchand et al. 2004, 2006; Pérez et al. 2021). However, few studies were interested in the variability of dissolved organic matter (DOM) quantity and quality in mangrove soils. Marchand et al. (2004) highlighted that dissolved organic carbon (DOC) increases with depth in Guianese mangrove soils, in contrast to particulate organic carbon (POC). In addition, fluorescence/DOC ratios and the hydrophobic contents of DOC increase with depths, indicating a more refractory DOM compared to the upper layer (Marchand et al. 2006). This suggests that the DOM is produced in the upper layers before migrating in a refractory phase at depth. DOM can indeed migrate with depth, notably trough convection processes, but can also be transported to adjacent ecosystems with phenomenon such as tidal flushing and pumping. Recent studies, including Xiao et al. (2023), highlight the key role played by tidal pumping in controlling fluxes between mangrove groundwater and coastal waters, particularly in terms of DOM encompassing both coloured dissolved organic matter (CDOM) and fluorescent dissolved organic matter (FDOM). Accordingly, DOM from mangroves exhibits typical chemical signatures and retains their recalcitrance even after being exported to adjacent ecosystems (Knoke et al. 2024). Remarkably, mangroves begin to emerge in the literature as substantial contributors, accounting possibly for more than 10% of terrestrial DOM fluxes to the oceans, despite occupying less than 0.1% of the Earth’s continental surface area (Dittmar et al. 2006).

To characterize the dynamics and sources of DOM, their optical properties, which include CDOM and FDOM, have been widely used in the literature. In coastal environments, these properties can be investigated both in the water column (Coble 1996; D’Andrilli et al. 2022; Dias et al. 2020) and in mangrove soil porewater (Chen & Hur 2015; Knoke et al. 2024; Wang et al. 2013).

Fluorescence techniques, such as excitation-emission matrix (EEM) fluorescence combining with parallel factor analysis (PARAFAC), are sensitive enough to differentiate between different types of fluorophores and therefore sources of DOM (Bro 1997; Coble 1996; Murphy et al. 2011). Optical properties reveal that wetland DOM dynamics are driven by source variability (autochthonous or allochthonous) and environmental transformations, including hydrology, primary production, and OM degradation, which reflect key ecosystem processes (Chen et al. 2010, 2013; Jaffé et al. 2008; Maie et al. 2005; Martín et al. 2023).

Despite these studies, there is currently a lack of information regarding the identification of a specific signature for DOM unique to mangrove forest ecosystems. Furthermore, the determination of DOM types associated with each mangrove species has not been thoroughly explored, which is crucial for assessing the role of mangroves in the biogeochemistry of subtropical ecosystems. Such knowledge would enable the determination of the proportion of autochthonous DOM from mangroves and exported to adjacent ecosystems and therefore better constrain the mangrove carbon cycle. A comprehensive understanding of these aspects is important in the context of monitoring and preserving mangroves.

The main objective of this study is to explore the quantity, composition, and origin of DOM in mangrove soil porewater using the optical properties of DOM. We seek to identify distinctive signatures attributed to different mangrove species contributions. To this end, we collected soil and porewater samples at four sites, under two species (*Rhizophora (R.) apiculata* and *Bruguiera (B.) gymnorhiza*) at two different growth rates, young and mature. Our hypothesis is that DOM concentration evolves along a gradient with depth and differs according to the two conditions: species and stage of mangrove development, with the expectation that OM will be more abundant under mature trees than under young ones.

## Material and Methods

### Study site

**Fig. 1:**
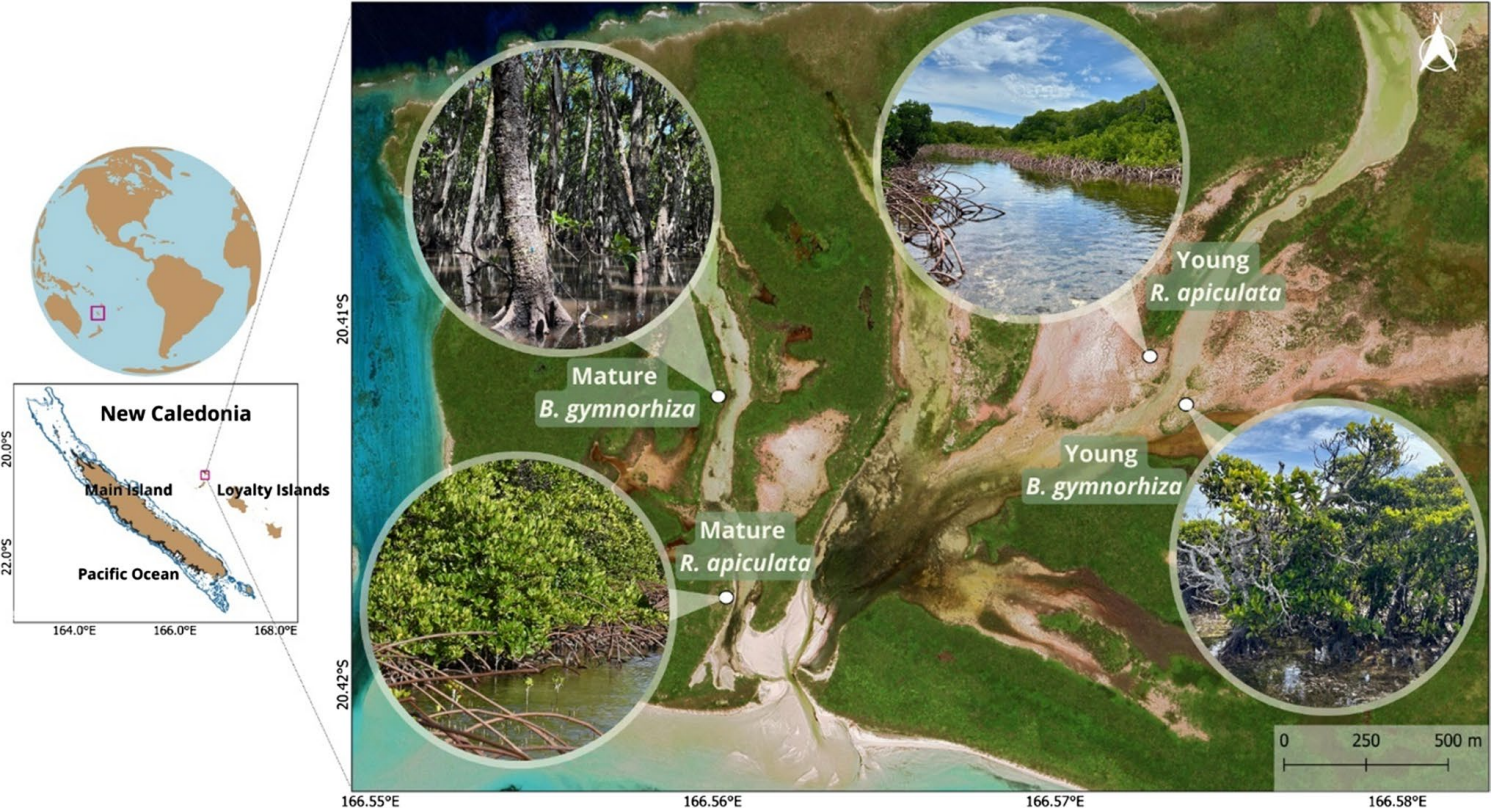
Map of the study area showing the four sites corresponding to the state of growth (mature and young) and the species (*B*. *gymnorhiza* and *R.* *apiculata*) (QGIS 3.28.6, Google earth, December 2024)

Located in the southwestern Pacific Ocean, New Caledonia is an archipelago composed of a main island named “Grande Terre” and additional islands named Loyalty Islands (Fig. 1). It hosts the world’s second-largest tropical oligotrophic lagoon, surrounded by an extensive 1800-km2 barrier reef (Benavides et al. 2018; Payri et al. 2018). Recognized for its remarkable biodiversity (Roberts et al. 2002), the region earned UNESCO World Heritage status in 2008 (United Nations Educational, Scientific and Cultural Organization).

Within the low-lying atoll carbonate of Ouvéa, the pristine tropical mangrove forest located at the northern end of the island (20.420°S, 166.562°E) stands as a remarkable natural feature (Fig. 1). This mangrove is characterized by a complex network of channels, with a unique channel opening into the Ouvéa lagoon.

Geologically, the atoll consists primarily of an uplifted paleoreef fringing system from the Pliocene/Quaternary periods (Maurizot et al. 2020). Its inherited rocks consist mainly of non dolomitic and partly aragonitic biodetritic and bioconstructed limestones. The atoll also hosts a karstic system, with sinkhole, pit, and caves, the extent of which is unconstrained (Maurizot et al. 2020). On the northern side of the island, a mangrove, protecting it from the Pacific Ocean, and these carbonates are present in the form of interglacial terrace ranging from + 8 up to + 22 m above sea level high (Mörner 2020). The mangrove develops directly at the expanse of the limestome but also from carbonated sands, with characteristics likely similar to those of the lagoon (dominated by aragonite and calcite) (Maurizot et al. 2004). The mangrove substrate has a granulometry mainly dominated by silt and sand and is relatively poor in clays (Online Resource 1), leading to a medium porosity promoting the fluid circulation and water exchange, except in the lower section of the carbonated sands (> 30 cm of depth), where the clay fraction increases, likely favouring the water retention. Additionally, the presence of seagrass beds in sandy sediments in adjacent areas and within mangrove creeks adds an important ecological dimension, providing nursery zones for many species such as juvenile lemon sharks and contributing to sediment stabilization.

This mangrove is subject to a semi-diurnal tidal cycle. The hydrodynamic behaviour of this mangrove is influenced by a narrow tidal inlet connected to the lagoon, which causes a delay in water exchange. Monitoring reveals that, on average, the water level in the mangrove area is relatively low compared to the open lagoon due to its semi-enclosed nature (Online Resource 2). During high tide, the water level inside the mangrove channel is 20 cm lower than outside. On the other hand, the difference at low tide is 40 cm, as the water is retained longer inside the mangrove because the flow capacity of the narrow channel is limited.

Preserved from the impacts of anthropogenic activities, external terrigenous sediments and organic inputs, the Ouvéa mangrove holds immense cultural value, designated as a taboo area by the local communities. Restrictions on human access to this mangrove contribute to the preservation of its pristine state.

The Ouvéa mangrove hosts various mangrove species at different growth stages, young and mature, with a notable dominance of *R. apiculata* and *B. gymnorhiza*. The coexistence of these two species creates diverse habitats, supporting a complex and dynamic biodiversity.

Four sampling sites were selected for the study, each featuring a specific dominant mangrove species at a distinct growth stage, young or mature, to investigate the influence of species and age of mangrove on the composition of OM. This mangrove lacks a watershed, with water input resulting from rainfall and tidal seawater exchange. The first site is labelled “MR” for monospecific stands of mature *R. apiculata* mangrove (Fig. 1). The second site named “MB” consists of a mature monospecific stand of *B. gymnorhiza* forest. The geomorphology of this last site includes a basin with several dozens of centimetres of loss of altitude, likely resulting from the carbonated substrate dissolution, in which water flow appears limited with the surrounding ecosystem. The third and fourth sites are made up of young mangroves, with “YR” signifying a monospecific stand of young *R. apiculata* mangrove. Finally, the last site, “YB,” is characterized by a mix of young mangroves, with a dominant stand of *B. gymnorhiza* and some *R. apiculata*.

### Field sampling

Samples were collected in May 2023. For each site, triplicate of 30-cm-long soil cores were collected using a manual corer, spaced approximately 20 m apart from each other. The sampling was conducted at low tide to ensure optimal access. Soil cores were cut into three sections of 10 cm along the depth with a Teflon spatula. Additional soil samples were collected from the rhizosphere of two plant species, mature *B. gymnorhiza* and mature *R. apiculata* in November 2024 for mycorrhizal analysis*.* Five samples were collected for each species. Fifty grams of rhizospheric soil and roots of each study site were sampled. The soil was sampled by first digging a pit of 30-cm diameter and 10-cm depth, close to the plant stems to have access to the roots. Soil was then collected from the border of the pits in order to obtain a sample representing a vertical cross section across the root zones of these plants.

Physicochemical parameters, including pH, temperature, and redox potential (Eh), were conducted using specific sensors. pH measurements were performed using a 3320 WTW® SenTix® 81 glass electrode calibrated with standards (pH 4, pH 7, pH 10) prior to measurements, while redox potential was measured using a SenTix® ORP WTW® pH315i electrode checked daily using a standard (220 mV).

Porewater was immediately extracted from the three different depths (0–10 cm, 10–20 cm, 20–30 cm) using soil moisture samplers (Rhizon® MOM- 10 cm—Rhizosphere) with a 0.2-µm pore size, inserted into soil sections. A 10-mL pressurized syringe was connected for extraction. Salinity was measured using a refractometer (ATC) on the collected porewater samples. Then, porewater was passed through pre-combusted (450 °C, 4 h) 25-mm glass filters (Whatman GFF, 0.7 µm) for analysis of DOC, CDOM, and FDOM concentrations. Subsequently, porewater was stored under appropriate conditions for future analyses. Regarding COD analysis, the filtered samples were stored in the dark at 4 °C in pre-cleaned 24-mL glass tubes (Wheaton) equipped with Teflon/silicone septa. To ensure sample preservation, these tubes were acidified using HCl (pH < 2). For DOM analysis, the extracted porewater (10 mL) was transferred to glass vials previously cleaned with 10% HCl, rinsed three times with Milli-Q water, and combusted (450 °C, 6 h) before storage at 4 °C in darkness. The sediments samples were kept at − 20 °C.

### Analytical methods

#### Total and dissolved organic carbon (TOC and DOC)

TOC and DOC concentrations were determined using a Shimadzu TOC-VCSH carbon analyser, following the high-temperature catalytic oxidation method described by Suzuki et al. (1992). TOC was analysed using a Solid Sample Module, while DOC was measured as non-purgeable organic carbon (NPOC). The accuracy of the DOC measurements was better than 60 μg L^−1^. Blanks and standard solutions were analysed every 10 samples; all quality control replicates were within a 10% error. Samples were analysed in triplicate to ensure precision.

#### Coloured dissolved organic matter (CDOM)

CDOM measurements were carried out at room temperature using a Shimadzu UV- 1700® spectrophotometer, scanning the range from 200 to 700 nm with a step size of 1 nm. A Milli-Q water blank was used as a control, and data acquisition followed the method described by Coble et al. (1998).

Spectral slopes (*S*_275–295_ and *S*_350–400_), the *S*_*R*_ ratio, and the *E*_2_/*E*_3_ index were calculated following the methods described by Helms et al. (2008), Hansen et al. (2016), and Rocha et al. (1999).

#### Fluorescent dissolved organic matter (FDOM)

Fluorescence analysis was performed using a PERKIN ELMER LS 55 spectrofluorometer as already described by Martias et al., (2018) and Dupouy et al. (2020). Briefly excitation wavelengths ranging from 200 to 500 nm were used, with measurements taken every 5 nm for emission wavelengths between 280 and 550 nm. The excitation light source consisted of a 20-kW xenon flash lamp coupled with a Monk-Gillieson monochromator. Before analysis, the samples were brought to room temperature (20 °C) and transferred to a quartz cell with a 2-mm optical path length that had been pre-washed with 10% HCl. Prior to each analysis, the cell was rinsed three times with 10% HCl, then three times with fresh Milli-Q water, and, if the sample volume allowed, three times with the sample itself. The asymmetric cell was positioned so that the 2-mm-long side faced the excitation beam, while emission was collected from the 1-cm optical side. Milli-Q water blanks were used to check cuvette cleanliness every three samples.

The excitation-emission matrix spectroscopy (EEMS) technique was employed, allowing for the generation of a 3D map representing fluorophore composition (Coble 1996, 2007). The EEM data were analysed using parallel factor analysis (PARAFAC) to identify dominant fluorophore groups (Bro 1997). PARAFAC was analysed using the ProgMEEF software (Mediterranean Institute of Oceanography (MIO)—Université de Toulon, 2018) through the MATLAB R2017a software.

Prior to interpretation, all data were normalized to Raman units (r.u) by dividing the EEMs by the Raman scattering peak of pure water at *Ex*/*Em* = 275/303 (Coble 1996). This normalization step eliminated the influence of Raman and Rayleigh scattering bands and was performed according to the method described by Zepp et al. (2004). Such normalization ensures a direct comparison of fluorescence intensities across studies, regardless of the instrument used (Gamrani et al. 2023). Then, the CORCONDIA (CORe CONsistency DIAgnostic) algorithm was used to identify the main fluorophore groups. This algorithm calculates a percentage value, with numbers exceeding 60% considered as the optimal number of components (Zhao 2011). These components were compared with existing literature. Various fluorescence indexes such as BIX (biological index) (Parlanti et al. 2000) and HIX (humification index) (McKnight et al. 2001) were calculated from the 3D fluorescence spectra to characterize the origin of material.

The relative abundance of each fluorescent component was calculated as the ratio of its fluorescence intensity to the total fluorescence intensity of all components, as described by Santin et al. (2009). For example, the relative abundance of component C1 is expressed as follow:

2 $$C1\left(\%\right)=\frac{florescence\;intensity\;of\; CI}{florescence\;intensity\;of\; CI+C2+C3+C4}\times100$$

#### Mycorrhizal analysis

Roots of each sample were stained using trypan blue following the method of Phillips and Hayman (1970) with minor modifications as described by BŁaszkowski et al. (2006). For each sample, 10 stained root fragments (1 cm long) were mounted on slides in glycerol to assess arbuscular mycorrhizal fungi (AMF) colonization under a compound microscope (Olympus BX 50, 200 × magnification), following the method of Trouvelot et al. (1986). We observed the presence/absence of hyphae, vesicules and arbuscules of AMF.

### Statistics

Statistical analyses were performed using the R software (v.4.0.3). The analysis included descriptive statistics, correlation matrix assessment, and comparison tests between sites and species. A significance level of *p* < 0.05 was chosen to determine statistical significance, with *p* > 0.05 indicating non-significance.

To evaluate statistical differences between sites and stations, the Kruskal–Wallis non-parametric test was employed since the assumptions of normality and homoscedasticity were not met. If significant differences were detected through this initial test, pairwise multiple comparisons were subsequently conducted using the Wilcoxon test to determine specific significant differences.

## Results

### Variability of soils physicochemical parameters

**Fig. 2:**
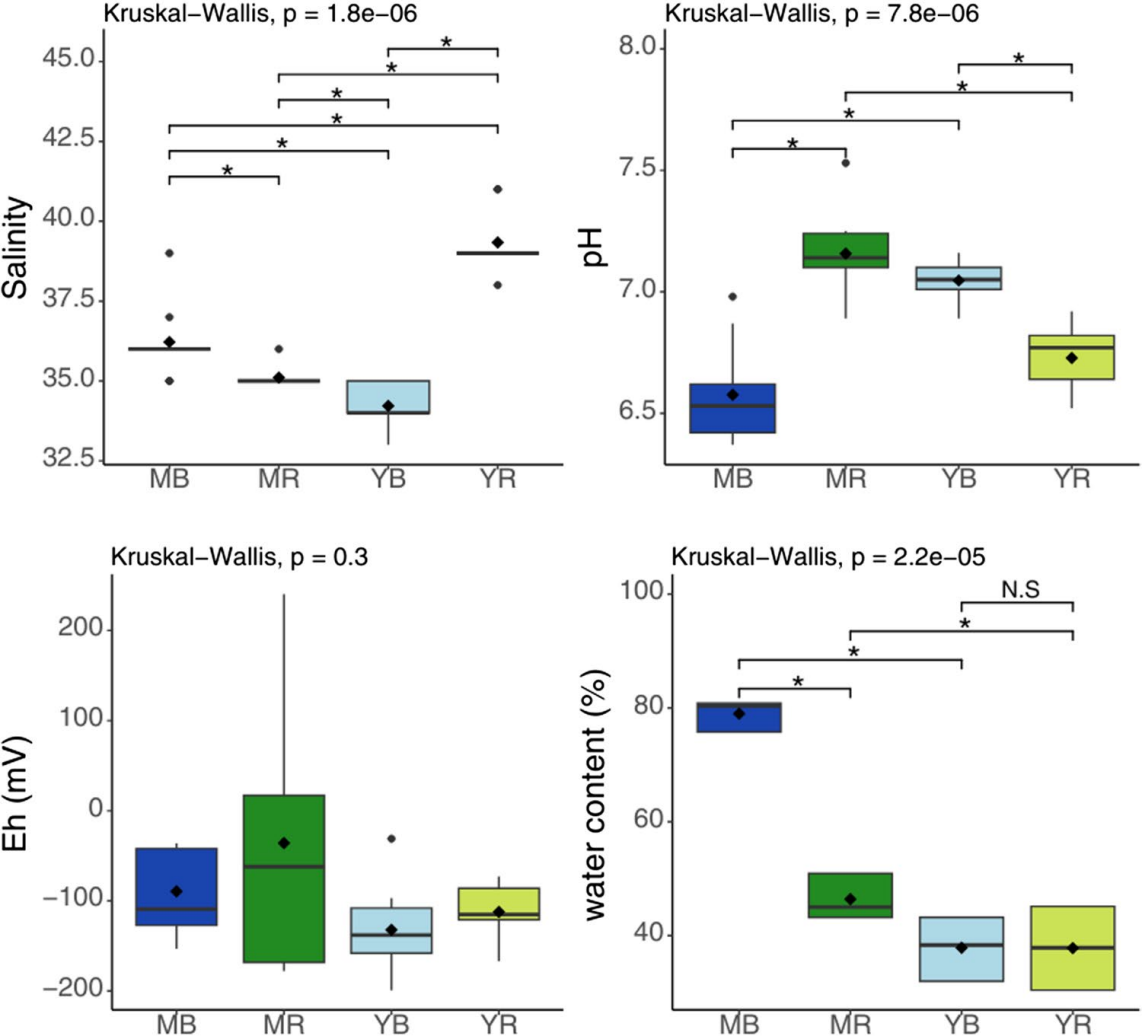
Boxplot of in situ physico-chemical parameters (salinity, pH, Eh, and water content) determined in sediment porewaters from mature *B. gymnorhiza* (MB), mature *R. apiculata* (MR), young *B. gymnorhiza* (YB), and young *R. apiculata* (YR) (*n* = 9). The asterisk represents significant differences with *p*-value < 0.05, and N.S means no significant differences

Porewater salinity (Fig. 2) ranged from 33 at young *B. gymnorhiza* (YB- 2; 10–20 cm) to 41 (‰) at young *R. apiculata* (YR- 2; 20–30 cm). Statistical analyses indicate that the salinity values are significantly different between sites (*p* < 0.05; Kruskal–Wallis). In terms of depth variation, salinity generally did not vary significantly within the same site, except at the young *B. gymnorhiza* site (YB), where a decrease in salinity was observed between the first 10-cm and the 20–30-cm-depth sections.

pH values greatly varied between sites (Fig. 2), with a minimum of 6.37 at mature *B. gymnorhiza* (MB- 3; 20–30 cm) and a maximum of 7.53 at mature *R. apiculata* (MR- 2; 20–30 cm). Non-parametric statistical tests revealed significant differences in pH across all sites. The mean pH (*n* = 9) at mature *B. gymnorhiza* (6.58 ± 0.21) was significantly different from that of mature *R. apiculata* (7.16 ± 0.18) and young *B. gymnorhiza* (7.05 ± 0.08). Additionally, pH values beneath mature *R. apiculata* were significantly different from that beneath young *R. apiculata* (6.73 ± 0.13), and the pH values at young *B. gymnorhiza* site were significantly different from those at young *R. apiculata* site (*p* < 0.05; Kruskal–Wallis). No significant differences in pH values were observed with the depth (*p*-value > 0.05). However, distinct trends emerged: at the MR, YB, and YR sites, pH values tended to increase with depth, while at the MB site, pH values showed a tendency to decrease with depth. Although these trends were not statistically significant, they suggest potential site-specific factors influencing pH profiles.

The redox potential ranged from − 199 mV (YB- 3; 20–30 cm) to + 240 mV (MR- 3; 0–10 cm) across the different sites. Specifically, the average redox values at each site along the entire sediment core were as follows: MR exhibited an average Eh value of − 35.7 ± 143 mV, YB showed − 132 ± 48.7 mV, YR had − 112 ± 31.5 mV, and MB presented a lower average Eh value of − 89.6 ± 47.3 mV. Statistical tests indicated no significant differences in Eh values both intra-site and inter-site (*p* > 0.05; Kruskal–Wallis). No significant differences with depth were observed across the four sites. Nonetheless, distinct trends were noted: at the MR, YB, and YR sites, the redox values generally increased with depth, suggesting a deeper oxidation layer. On the contrary, at the MB site, redox values tended to decrease with depth.

The soil water content in mangroves varies significantly among the four mangrove stands, with the highest mean water content found under mature *B. gymnorhiza* (79.0% ± 2.41), followed by 46.4% ± 3.50 under mature *R. apiculata*. In soils under young trees, water content does not differ significantly, remaining around 37% for both young stands (Fig. 2).

### Organic matter distribution

#### Variability of carbon quantity in mangrove soils porewater

**Fig. 3:**
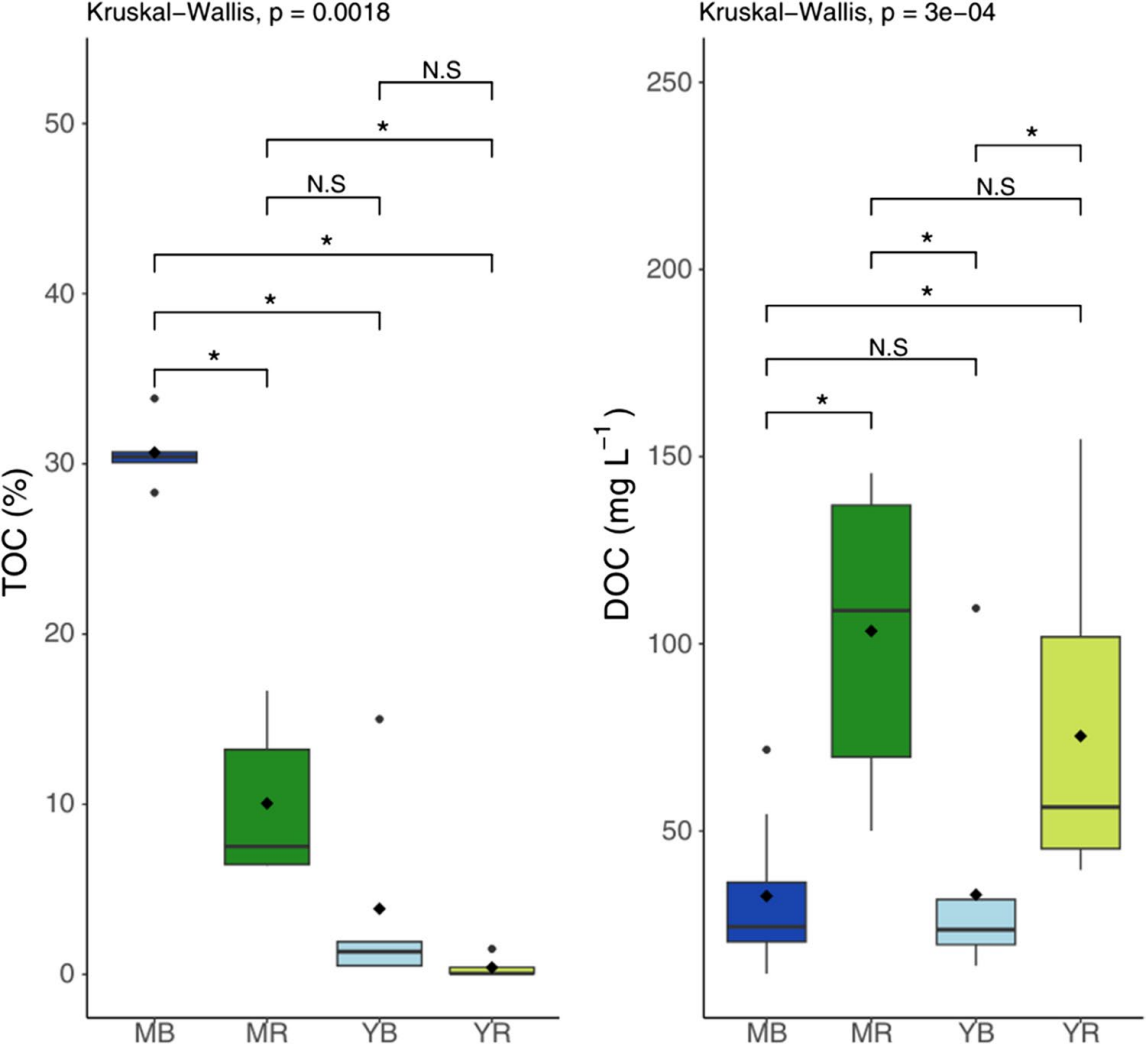
Box plots of TOC (%) and DOC concentration (mg L ^−1^) in soil beneath mature *B. gymnorhiza* (MB), mature *R. apiculata* (MR), young *B. gymnorhiza* (YB), young *R. apiculata* (YR). The asterisk represents significant differences with *p* -value < 0.05, and N.S means no significant differences

The percentages of solid organic carbon in mangrove soils are higher under mature trees; however, a significant difference exists between species, with the highest TOC content under mature *B. gymnorhiza* (30.7% ± 2.0), followed by mature *R. apiculata* (10.0% ± 4.65). The lowest contents are found under young trees, with 3.85% ± 6.26 under *B. gymnorhiza* and the lowest at 0.4% ± 0.7 under young *R. apiculata*. The trends observed for solid organic carbon (TOC) in mangrove soils do not seem to correspond to that observed for dissolved organic carbon (DOC); however, distinct profiles seem to emerge depending on the species (Fig. 3).

DOC concentrations varied considerably across sites, with a similar trend within species (Fig. 3). *Rhizophora apiculata* sites had higher DOC concentrations than *B. gymnorhiza* sites, particularly in mature *R. apiculata* (103 ± 38.3 mg L^−1^) and to a lesser extent in young *R. apiculata* (75.4 ± 40.1 mg L^−1^). However, no significant differences were detected between growth stages whatever the species. *Bruguiera gymnorhiza* showed an average DOC concentration of 32.6 ± 19.2 mg L^−1^ in mature stands (MB) and 32.9 ± 29.4 mg L^−1^ in younger stands (YB). Regarding depth variations, no significant differences in DOC concentration (mg L^−1^) were observed within each site.

#### Variation of DOM quantity and quality beneath mangrove species

The average absorption coefficient at 350 nm (*a*_350_), a proxy for CDOM quantity, varied from 13.6 ± 3.91 m^−1^ at YB to 65.1 ± 46.0 m^−1^ at YR (Table 1). The average values across the entire sediment core at each site were 34.5 ± 32.9 m^−1^ (YR), 22.6 ± 6.4 m^−1^ (MB), 19.2 ± 5.75 m^−1^ (MR), and 15.4 ± 6.65 m^−1^ (YB). Statistical tests revealed no significant differences in *a*_350_ between sites or within sites. Regarding depth variations, no significant differences or trends were observed across the four sites (*p* > 0.05; Kruskal–Wallis).

**Table 1:** Mean ± standard deviation of CDOM parameters (*a*_350_, *a*_442_, *S*_275–295_, *S*_350–400_, *S*_*R*_, *E*_2_/*E*_3_) determined in sediment porewater from mature *B. gymnorhiza* (MB), mature *R. apiculata* (MR), young *B. gymnorhiza* (YB), and young *R. apiculata* (YR)

Sample	Depth (cm)	*a*_350_ (m^−1^)	*a*_442_ (m^−1^)	*S*_275–295_	*S*_350–400_	*S*_*R*_	*E*_2_/*E*_3_
MB	0–10	**19.6** $\pm$6.78	**4.68** $\pm$1.57	**0.017** $\pm$0.000	**0.015** $\pm$0.000	**1.11** $\pm$0.35	**4.33** $\pm$0.12
	10–20	**22.4** $\pm$4.65	**4.76** $\pm$0.53	**0.019** $\pm$0.002	**0.016** $\pm$0.000	**1.13** $\pm$0.09	**4.81** $\pm$0.26
	20–30	**25.8** $\pm 8$.14	**5.22** $\pm$1.13	**0.020** $\pm$0.001	**0.017** $\pm$0.001	**1.18** $\pm$0.19	**5.95** $\pm$0.94
MR	0–10	**23.2** $\pm$7.98	**12.3** $\pm$8.48	**0.025** $\pm$0.003	**0.010** $\pm$0.004	**2.87** $\pm$1.30	**6.27** $\pm$0.54
	10–20	**18.8** $\pm$4.45	**9.36** $\pm$4.79	**0.023** $\pm$0.003	**0.011** $\pm$0.003	**2.14** $\pm$0.63	**6.24** $\pm$0.97
	20–30	**15.4** $\pm$1.51	**6.44** $\pm$0.60	**0.023** $\pm$0.003	**0.012** $\pm$0.002	**1.98** $\pm$0.66	**6.82** $\pm$0.28
YB	0–10	**13.6** $\pm$3.91	**4.60** $\pm$2.42	**0.019** $\pm$0.001	**0.014** $\pm$0.003	**1.37** $\pm$0.32	**5.14** $\pm$0.77
	10–20	**20.3** $\pm$9.90	**9.21** $\pm$9.19	**0.018** $\pm$0.004	**0.011** $\pm$0.005	**1.76** $\pm$0.54	**4.58** $\pm$1.65
	20–30	**12.2** $\pm$2.88	**3.99** $\pm$0.66	**0.022** $\pm$0.001	**0.013** $\pm$0.000	**1.63** $\pm$0.13	**5.47** $\pm$0.02
YR	0–10	**20.7** $\pm$3.93	**8.06** $\pm$1.84	**0.018** $\pm$0.001	**0.012** $\pm$0.000	**1.50** $\pm$0.12	**4.84** $\pm$0.24
	10–20	**65.1** $\pm$46.0	**44.1** $\pm$34.8	**0.015** $\pm$0.007	**0.008** $\pm$0.004	**2.00** $\pm$0.41	**3.96** $\pm$1.47
	20–30	**17.8** $\pm$8.90	**7.21** $\pm$4.73	**0.018** $\pm$0.001	**0.013** $\pm$0.001	**1.40** $\pm$0.37	**6.00** $\pm$0.76

Bold values indicate the mean values, while standard deviations are presented in regular font

The average absorption coefficient at 442 nm (*a*_442_) (Table 1), which can be used as a proxy for biological activity, varied significantly across sites. The average *a*_442_ values across the entire soil core at each site were as follows: 19.8 ± 25.4 m^−1^ at YR, 9.37 ± 5.50 m^−1^ at MR, 6.04 ± 4.44 m^−1^ at YB, and 4.89 ± 1.03 m^−1^ at MB. The highest recorded value of *a*_442_ was 44.1 ± 34.8 m^−1^ at YR, while the highest for *B. gymnorhiza* was 9.21 ± 9.19 m^−1^ at YB. Statistical analysis revealed significant differences between species (*p* < 0.05; Kruskal–Wallis). Regarding depth variations, no significant differences in *a*_442_ were observed within each site.

The average spectral slopes *S*_275–295_ (Table 1) varied among sites. The means across the entire sediment core were 0.017 ± 0.004 for YR, 0.019 ± 0.002 for MB, 0.020 ± 0.003 for YB, and 0.024 ± 0.003 for MR. The highest average spectral slope was observed at the MR site (0.024 ± 0.003), while the lowest was at the YR site (0.017 ± 0.007). Statistical analysis revealed that the *S*_275–295_ at YR were significantly lower than those at MR (*p* < 0.05; Kruskal–Wallis) suggesting the presence of higher molecular weight molecules at YR compared to MR. No significant differences in spectral slopes were observed between the MB and YB sites, with values being around 0.020. In terms of depth variations, no significant differences in spectral slope values were observed within each site across the soil core, suggesting no substantial change within the soil column.

The slope ratio (*S*_*R*_), an indicator of molecular weight and photodegradation, varied significantly among sites (Table 1). The average *S*_*R*_ values across the entire sediment core at each site were the highest at MB with 1.14 ± 0.11, followed by YB with 1.59 ± 0.36, and YR with 1.63 ± 0.35, and finally MR with 2.34 ± 0.89. Significant differences in *S*_*R*_ between MB and MR (*p* < 0.001; Kruskal–Wallis) were observed, as well as between MB and YB (*p* < 0.01; Kruskal–Wallis). The *S*_*R*_ was significantly higher at MR compared to MB and YB, indicating a presence of higher molecular weights and greater photodegradation at the MR site. However, no significant difference was observed between the two *R. apiculata* sites (YR and MR). In terms of depth variations, no significant differences or trends were detected within the sediment cores at any of the sites.

The *E*_2_/*E*_3_ ratio, which estimates the size and aromaticity of humic substances, varied also significantly among sites (Tab1). The average *E*_2_/*E*_3_ values across the entire sediment core at each site were 6.45 ± 0.64 at MR, 5.07 ± 0.10 at YB, 5.03 ± 0.87 at MB, and 4.93 ± 1.22 at YR. The *E*_2_/*E*_3_ ratio at the MR site was significantly higher compared to the other three sites (*p* < 0.05; Kruskal–Wallis). In contrast, the lowest *E*_2_/*E*_3_ values were observed at the YR site, with a minimum value of 3.96 ± 1.47. No significant differences or clear trends in the ratio were observed with depth at any of the sites.

#### EEM PARAFAC components of FDOM

**Fig. 4:**
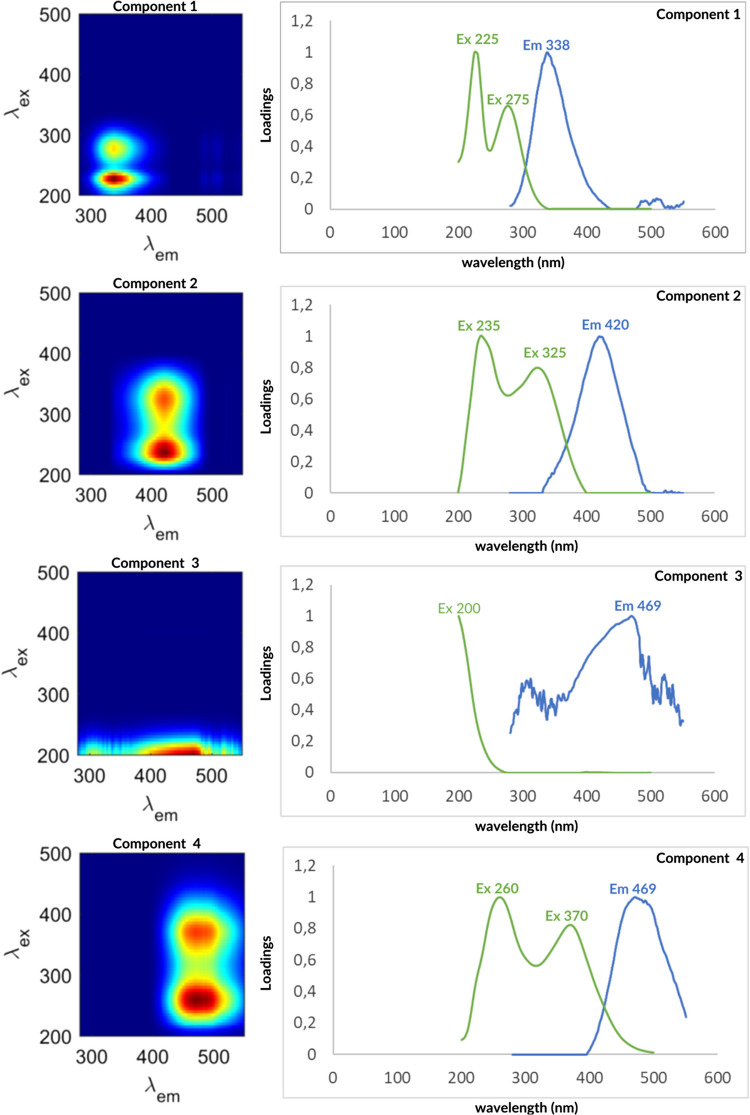
Contour plot of the four fluorescent components identified from PARAFAC model. C1 identified as tryptophan-like component, C2 as a humic-like fluorophore, C3 as a fulvic acid, and finally C4 as humic-like

In this study, four fluorescent components (C1–C4) were validated by PARAFAC analysis, using 36 EEMs from soil porewater. The four-contour plots identified in this study are represented in Fig. 4 with their maximum excitation and emission. To determine the sources of these components, we compared their spectral characteristics with literature and OpenFluor database (Murphy et al. 2014), which resulted in significant spectral matches (Tucker’s correlation coefficient > 0.95) with previously documented spectra (Table 2). While different studies may have used various spectrofluorometers, the identification of fluorophores relies on their relative spectral characteristics, such as peak positions and overall trends, rather than on absolute fluorescence intensities, which cannot be directly compared.

**Table 2:** Identification of fluorophores and comparison of the spectral characteristic of the components identified in Ouvéa (May 2023) with those of other study uploaded on OpenFluor database with a spectral match of Tucker’s correlation coefficient > 0.95

Ouvea May 2023				Other study					
Comp	*λ*_*Ex*_ (nm)	*λ*_*Em*_ (nm)	Type	Comp	*λ*_*Ex*_ (nm)	*λ*_*Em*_ (nm)	Reference	Type	Origin
C1	225(275)	338	Tryptophan-like	C4	274	335	Batista-Andrade et al. (2023)	Tryptophan-like	Decomposition of aquatic plant biological wastewater treatment
				C5	275	345	Logozzo et al. (2023)	Protein-like	Autochthonous; tryptophan
				C5	< 250	348	Murphy et al. (2011	Protein-like, tryptophan	
				C5	280	340	Moona et al. (2021)	Protein-like	Microbial-origin, “T” peak
				C5	270	< 350	Cawley et al. (2012	Protein-like	similar to the amino acid tryptophan
				C3	280	335	DeFrancesco and Guéguen (2021)	Protein-like Tryptophan-like	Recent biological production Amino acids or protein-like material
				C5	280	342	Peleato et al. (2017)	Tryptophan/protein-like	
				Region II	200–250	330–380	Chen et al. (2003)	Aromatic Protein	
				Region IV	250–340	280–380	Chen et al. (2003)	Soluble microbial by product-like	Tryptophan
C2	235(325)	420	Terrestrial or marine humic-like	C1	< 250 (320)	422	Yamashita et al. (2011)	Peaks A and C	Terrestrial humic-like
				C1	270(320)	411	Amaral et al. (2020)	Humic-like	Humic-like in aquatic environments
				M	312	380–420	Coble (1996)	Marine peak M	Marine-humic-like
				A	260	380–460	Coble (1996)	Humic-like	
				C1	240(310)	429	Garcia et al. (2015)	Peaks A and M	Terrestrial humic-like
				C2	330	400	Catalá et al. (2015)	Humic-like	
				C3	< 260(< 315)	421	Yamashita et al. (2008)	Marine peak M	Marine humic-like or microbial oxidized component
C3	200	469	Terrestrial material Fulvic acid	Region III	200–250	380–550	Chen et al. (2003)	Fulvic acid Region III	Fulvic-like material
				C1 Region III	< 240 < 250	436 > 380	Stedmon et al. (2003) Martín et al. (2023)	UV humic-like Fulvic substances	UV humic-like material Fulvic substances
C4	260(370)	469	Humic-like	C2	< 260(370)	475	Dainard et al. (2015)	Humic-like	Humic-like
				C3	250	480	Peleato et al. (2017)	Humic-like	Terrestrial humic-like
				C1	< 260(365)	475	DeFrancesco and Guéguen (2021)	Peaks A and C	Terrestrial humic-like
				C2	250(350)	470	Sharma et al. (2017)	Humic-like	Humic-like
				C2	265(370)	464	Yamashita et al. (2011)	Humic-like	Humic-like components
				C4	260(305)	404	Chen et al. (2018)	Humic-like	Marine humic-like
				C2	260(365)	480	Gamrani et al. (2023)	Humic-like	UV/visible humic-like
				C2	250(360)	468	Kida et al. (2021)	Humic-like	Plant, terrestrial humic-like

The spectral characteristics of the first component C1 were composed of two excitation maxima, with peak at 225 nm and the second at 275 nm with emission at 338 nm. The C2 and C4 components have been characterized as humic-like components (Tab. 2), with two excitations peaks (C2: 235(325) and C4: 260(370)) and one emission peak at 420 nm for C2 and at 469 nm for C4. The EEM spectral characteristics of the C3 component were composed of 200 nm excitation maxima and 469 nm emission peak. These wavelengths agreed with fulvic-acid (Chen et al. 2003) and UV-humic-like substances (Stedmon et al. 2011). Given that the spectrum exhibited characteristics that could be interpreted as noise, further analyses were conducted to minimize potential inner filter effects and for eliminating Rayleigh and Raman scatter peaks. Simulations were performed in PARAFAC, progressively increasing the Zepp correction up to 45-nm bands widths to reduce these interferences. Despite these adjustments, the component consistently exhibited a clear signature with weak excitation and an emission at 469 nm, further supporting its identification as a terrestrial material.

#### Distribution of FDOM components under mangrove species

**Fig. 5:**
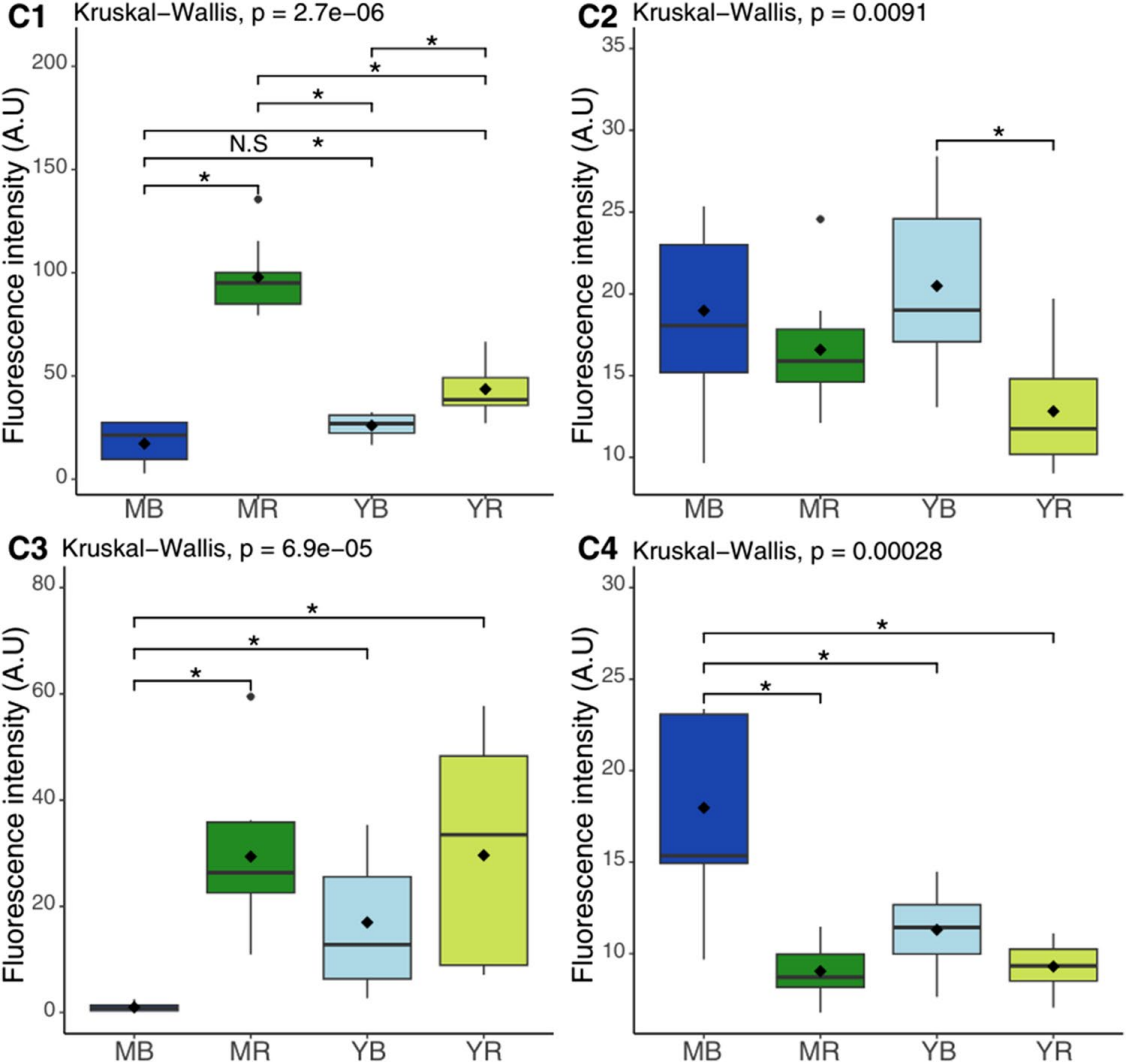
Box plots of DOM fluorescence of four components validated by PARAFAC (C1–C4) in the soil of the four sites: mature *B. gymnorhiza* (MB), mature *R. apiculata* (MR), young *B. gymnorhiza* (YB), and young *R. apiculata* (YR). The asterisk represents significant differences with *p*-value < 0.05, and N.S means no significant differences

The variability of fluorescence intensity of the four components with their spatial distribution among the two stages of mangrove species is shown in Fig. 5. Their progression with depth is presented in Online Resource 3.

The tryptophan-like component (C1) exhibits significantly higher abundance in the mature *R. apiculata* site. The fluorescence intensity of the humic-like components (C2 and C4) is higher in the *B. gymnorhiza* sites compared to the *R. apiculata* sites. Specifically, the C2 compound is significantly lower at the YR compared to YB, indicating a very low humic-like contribution at YR compared to the YB site. The contribution of C4 in MB is significantly higher than in the other three sites. C3 component (fulvic acid) appears to be absent in the MB site when compared to the other three sites.

The contribution of fluorophores in FDOM was quite heterogeneous overall. We observed a similar trend for the *R. apiculata* sites, with a higher fluorescence intensity of the compound tryptophan-like C1, showing a predominant presence in mature *R. apiculata* and a lesser extent in young *R. apiculata*.

The *B. gymnorhiza* sites also shared common characteristics, with lower fluorescence intensity compared to those of the *R. apiculata* sites. The tryptophan-like compound C1 and the fulvic-acid compound C3 were lower at *B. gymnorhiza* sites, while the two humic-like compounds, C2 and C4, were higher compared to *R. apiculata* sites. A mixed trend was observed beneath young *B. gymnorhiza*: Fluorescence intensity was low for C1, higher for C2 and C4, and C3, also present at the *R. apiculata* sites, was detected as well.

At the MR site, the most prominent compound is tryptophan, followed by fulvic acid, and then the two humic compounds (C2 and C4). A similar trend is observed at the young *R. apiculata* (YR) site, with differences in fluorescence intensity. The humic compounds (C2 and C4) are the least abundant in both sites. In contrast, at the *B. gymnorhiza* site, there is an opposite trend, with a majority of humic origin compounds (C2 and C4), followed by a high intensity of tryptophan (C1). The fulvic acid compound (C3) is absent from the mature *B. gymnorhiza* site.

**Fig. 6:**
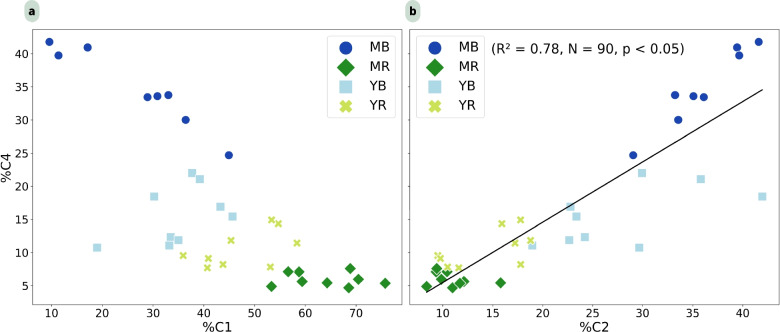
** a** Percentage distribution of PARAFAC component C4 (mangrove-derived humic-like) versus C1 (protein-like). **b** Percentage distribution of C4 versus C2 (coastal humic-like) across all samples, color-coded by mangrove stand type

Comparing the relative abundances of the components (C %), distinct differences were observed between the two species and their growth stages. Beneath *R. apiculata*, the relative abundance of C1 increases with the growth stages (Fig. 6a). Whereas for *B. gymnorhiza*, the proportion of C4 (%) becomes more pronounced with stand development stages (Fig. 6a). Figure 6b reveals a significant positive linear correlation between the relative abundances of C4 and C2 (%) (*R*2 = 0.78, *p* < 0.05). Furthermore, C2 was more prevalent at *B. gymnorhiza* sites, showing a similar gradient with age as C4–C1 relationship (Fig. 6a). Notably, mature *B. gymnorhiza* trees displayed a high C4/C2 ratio, while *R. apiculata* trees, particularly the mature ones, exhibited lower C4/C2 ratios (Fig. 6b).

Regarding the variations in pseudo-concentrations of fluorescent compounds with depth (Supplementary 3), no significant differences were observed across the four sites (*p* > 0.05; Kruskal–Wallis). However, although these differences are not statistically significant, trends appear to emerge depending on the compounds and the sites studied. For compound C1, an increase in pseudo-concentrations with depth was observed at both *B. gymnorhiza* sites, while an opposite trend was noted at the *R. apiculata* sites. Regarding compound C2, trends vary between the *B. gymnorhiza* sites. An accumulation with depth was observed for mature trees, unlike young trees, in which pseudo-concentrations decreased with depth.

#### Characterisation of DOM using indexes

**Fig. 7:**
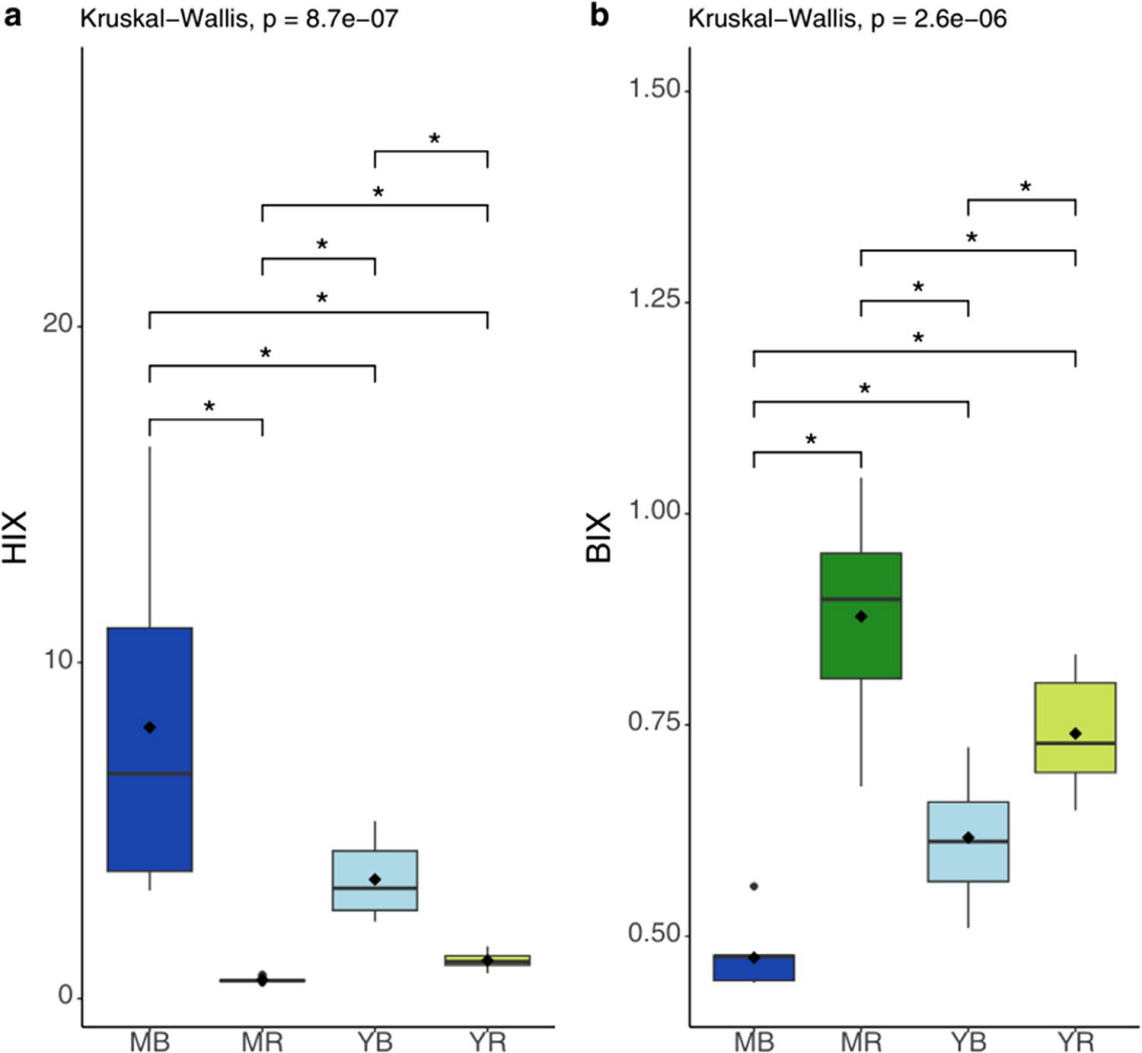
Boxplots representing the humification index (HIX) (**a**) and the biological index (BIX) (**b**) calculated for the four sites: mature *B. gymnorhiza* (MB), mature *R. apiculata* (MR), young *B. gymnorhiza* (YB), and young *R. apiculata* (YR). Note that the *y*-axes are not the same between the indices. The asterisk represents significant differences with p-value < 0.05

Figure 7 illustrates the boxplots of the humification index (HIX) and the biological index (BIX) across the four studied sites. The HIX and BIX values provide insights into the degree of humification and the biological activity within the sediment samples, respectively. The mean HIX (Fig. 7a) values across the entire sediment core at each site were 0.56 for MR, 1.14 for YR, 3.55 for YB, and 8.08 for MB. Analyses indicate significant differences in HIX values between sites (*p* < 0.05; Kruskal–Wallis). HIX values at the MB site are significantly higher than those at the other three sites, indicating a higher degree of humification.

The average BIX values range from a minimum of 0.47 at the MB site to a maximum of 0.88 at the MR site (Fig. 7b). Significant differences in BIX values were observed between the sites (*p* < 0.05; Kruskal–Wallis). The BIX values exhibited an inverse trend compared to those of the HIX indices, with values at MB significantly lower than at the other three sites. BIX values were higher in YB (0.62) than in MB (0.47), reflecting differences in biological activity. For *R. apiculata*, the inverse relationship was observed, with higher BIX values in MR (0.88) than in YR (0.74). Regarding depth variations, no significant trends were observed in either HIX or BIX values within the same site (Online Resource 4). The HIX and BIX values remained relatively stable across the depth profiles, indicating consistent humification and biological activity levels throughout the soil cores.

### Mycorrhizal analyses

**Fig. 8:**
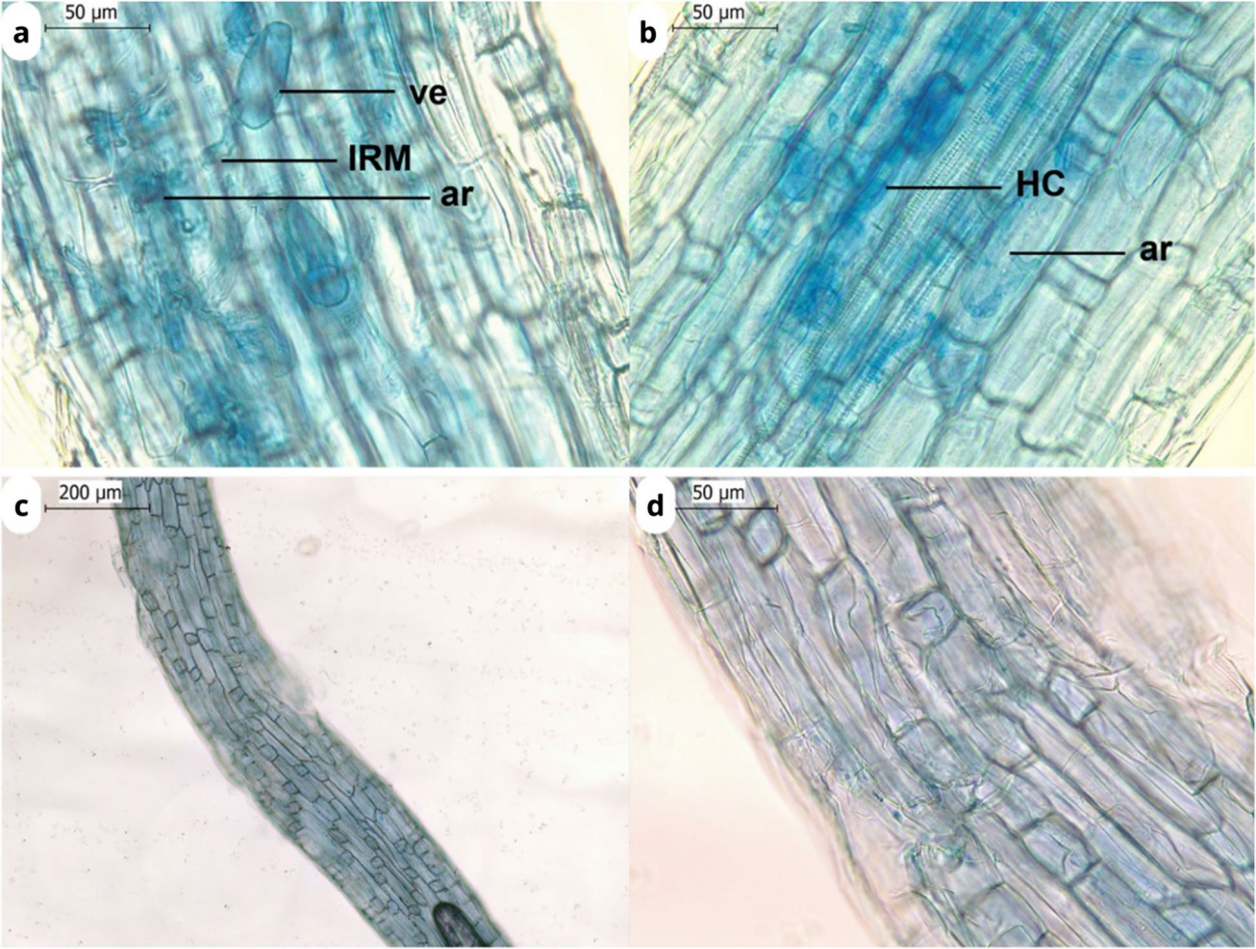
Roots of *B. gymnorhiza* (**a**, **b**) and *R. apiculata* (**c**, **d**) stained in Trypan blue. **a**. **b** Typical arbuscular mycorrhizal structures formed with *B. gymnorhiza* roots. ve: vesicule; IRM: intraradical mycelium; ar: arbuscule; HC: hyphal coil

Microscopy revealed the presence of arbuscular mycorrhizal fungal structures in the roots of *B. gymnorhiza* (Fig. 8a,b), but not in the roots of *R. apiculata* (Fig. 8c,d). The roots of *B. gymnorhiza* contained intra-radical mycelium, hyphal coils, and arbuscules. In contrast, no arbuscular mycorrhizal structures were detected in the roots of *R. apiculata* (Fig. 8c,d).

## Discussion

### Variability in the quality of DOM in mangrove soils across different mangrove stands

In Ouvéa mangrove, DOM can originate from (i) autochthonous production, i.e. decomposing wood and roots and senescent leaves (Maie et al. 2008; Shank et al. 2010), and microphytobenthos; (ii) allochthonous sources, such as coral exudates in coastal estuaries (Martias et al. 2018), or decomposing seagrass (Cawley et al. 2012) being brought in from the lagoon during the flow. There are no river inputs or effluents from anthropogenic activity (Fig. 1). In this context, our first objective was to characterize DOM sources using its fluorescence. Four components were identified.

The C1 component was more abundant in soils of *R. apiculata* stands compared to *B. gymnorhiza* (Fig. 7). It did not show any significant variation with depth (Online Resource 3), suggesting an autochthonous origin from decomposer organisms within the soil. This component is similar to an autochthonous tryptophan-like component, known as peak T (Coble et al. 1998), often linked to protein-like fluorescence from microbial sources (Stedmon et al. 2011; Murphy et al. 2011). We thus suggest that it is produced by soil microorganisms, rather than by microphytobenthos, which would typically be concentrated near the soil surface and disappearing with depth (Yin et al. 2016).

Conversely, C2 and C4 have been identified as terrestrial humic-like components (Table 2). These humic-like components are found in both terrestrial and costal environments because they originate from higher plants (Kida et al. 2021). These two compounds are positively correlated (Fig. 6b), suggesting a common source (Zhang et al. 2010). A component similar to C2 has been observed in an estuarine salt marsh in Spain, composed of the vascular plants (Santín et al. 2009). C4 has similar optical characteristics to a component observed in mangroves of Thailand (Kida et al. 2021). This component is present at all four Ouvéa mangrove sites, with notable concentrations in the *B. gymnorhiza* sites. This suggests that C4 could be a humic component characteristic of mangrove ecosystems. Indeed, this fluorescent component consists of two peaks: Peak A and Peak C (Stedmon et al. 2011; Yamashita et al. 2011). Previous studies have shown that these two peaks, A and C, can originate from senescent mangrove leaves, particularly from tannins (Maie et al. 2008; Shank et al. 2010). Maie et al. (2008) have demonstrated in their study that the diagenetic products of tannins can contribute to the presence of these peaks in tropical and subtropical estuaries. C2 and C4 are more prevalent at *B. gymnorhiza* sites than at *R. apiculata* sites (Fig. 6b), suggesting similarities with other coastal or terrestrial forests, in which DOM is produced through leaf litter decomposition (Moran et al. 1991). In contrast, the fluorescence signals of DOM in *R. apiculata* soils are less comparable to those of terrestrial ecosystems. *Bruguiera gymnorhiza*, like terrestrial vascular plants, have the ability to form arbuscular mycorrhizal associations, whereas *R. apiculata* does not (Fig. 8). These observations are in agreement with the study of Akaji et al. (2022, 2024), who observed arbuscular mycorrhizae in the *B. gymnorhiza* soil but not in *R. stylosa* soils. The similarities of the C2 compounds identified in *B. gymnorhiza* with those present in other estuarine ecosystems, such as in the Gulf of Cádiz, between the Mediterranean Sea and the Atlantic Ocean (Amaral et al. 2020), as well as in Liverpool Bay (Yamashita et al. 2011) could be explained by the presence of these associations with fungi. Symbiotic associations with arbuscular mycorrhizal fungi are common in the roots of vascular plants, although colonization rates can vary depending on the ecological habitat (Brundrett 2009). In coastal habitats, such as mangroves, fungal distribution tends to be patchy, but may play a role in the decomposition of OM (Arfi et al. 2012). It has been described that mycorrhizae can produce exoenzymes able to degrade tannin-protein complexes (Bending & Read 1996; Maie et al. 2008; Wu et al. 2003). We assume that *B. gymnorhiza*, through the presence of this symbiotic association, might be able to produce a major contribution to peaks A and C due to diagenetic products of tannins, present in compounds C2 and C4.

Based on its fluorescence characteristics, the C3 component exhibited an excitation maximum below 200 nm and an emission peak at 469 nm (Fig. 4; Table 2). This pattern aligns with the humic-like fluorophore in the ultraviolet region (peak A), as characterized by Coble (1996), Coble et al. (1998), and Yamashita et al. (2008). This component shows similarities to terrestrial fluorescent components and fulvic-like substances identified in previous studies (Table 2) as referenced in the OpenFluor database. Although it is close to the compounds identified as terrestrial material, it could also be produced by mangroves.

Identifying fluorescence signatures unique to mangrove-derived DOM is challenging, as both humic-like and protein-like fluorescence co-occur in the four mangrove stands. However, distinct trends appear between mangrove species. DOM in the soils of *R. apiculata* was characterized by a fulvic-like fluorescence (C3) and protein-like fluorescence (C1), with a clear contribution of microbial-autochthonous sources (Murphy et al., 2008; Stedmon et al. 2011; Yamashita et al. 2011). Conversely, DOM in the soils of mature *B. gymnorhiza* was characterized by humic-like fluorescence (C2 and C4), evidencing vascular plant sources. The presence of arbuscular mycorrhizal fungi in these soils is suggested to play a key role in DOM production from the autochthonous mangrove litter. However, the soil beneath the young *B. gymnorhiza* site exhibited a mixed trend. This site shows similarities to the *R. apiculata* sites, as evidenced by the presence of the C3 compound (fulvic acid), which was observed in both young and mature *R. apiculata* stands but not in the mature *B. gymnorhiza s*tand. This mixed trend may be explained by the fact that the young *B. gymnorhiza* stands consist of dying young *B. gymnorhiza* trees gradually being replaced by *R. apiculata*. To better assess DOM quality, various fluorescence indexes such as BIX (biological index) were studied (Parlanti et al. 2000).

In mature *R. apiculata* stands, the elevated biological activity index (BIX) (Fig. 7) and *a*_442_ coefficient, as well as a higher *E*_2_/*E*_3_ ratio, indicate higher biological activity (Huguet et al. 2009) and the presence of lower molecular weight molecules, evidencing fresher DOM. This suggests that *R. apiculata* soils may be more favourable to biological activity than those of *B. gymnorhiza*, and thus to DOM production as shown by the differences in DOC concentrations measured between the two stands (Fig. 3). In addition, this result could be explained by the fact that *R. apiculata* leaf litter decomposes faster than *B. gymnorhiza* (Ashton et al. 1999; Pradisty et al. 2021), due to different C:N ratios of mangrove leaves (Ashton et al. 1999; Mfilinge et al. 2002). Physico-chemical characteristics of the soils also differed between species and may explain these trends. In the mature *B. gymnorhiza* sites, the soil was the most acidic of Ouvea mangrove (Fig. 2). Such low pH may inhibit microbial activity, leading to slower decomposition of the litter as observed in soils with pH around 4 in the study of Wu et al. (2023), although the pH in our study sites was not as low (6.58 ± 0.21). This hypothesis is in line with statistical analysis (correlation matrix), a positive correlation was observed between pH and the C1 component (Online Resource 5) (Spearman’s correlation: 0.52, *p*-value < 0.05). In contrast, an inverse relationship was observed between pH and the C4 component (Spearman’s correlation: − 0.59, *p*-value < 0.05).

Consequently, DOM quality, based on their fluorescent properties, differed between stands. *Rhizophora apiculata*-DOM is optically characterized by higher *a*_442_ values, a higher BIX index, a lower HIX index, higher *S*_275–295_, and a greater proportion of fulvic-like and protein-like fluorescence than *B. gymnorhiza*-DOM. Arbuscular mycorrhizal fungi, associated with a specific mangrove tree, seem to play a key role in DOM quality, as well as the pH, which can be partly controlled by organic matter accumulation in mangrove soils, as detailed in the next section.

### Variability of organic matter quantity across mangrove stands

The accumulation of DOM in mangrove soils results from competing processes including production, decomposition, sorption, and transport through vertical gradients or tidal pumping (Kristensen et al. 2017). In Ouvéa, DOC concentrations were significantly higher beneath *R. apiculata* trees than beneath *B. gymnorhiza* trees regardless of the stage of development and despite lower soil organic matter content. This result may reflect enhanced DOM production beneath *R. apiculata* trees. We suggest that the high biological activity, as evidenced by the high BIX index described in the previous section, partly explains these higher DOC concentrations. In addition, beneath *R. apiculata* stands DOC values increased from the young to the mature stand, with values of around 75 mg L^−1^ and 103 mg L^−1^, respectively. This trend is consistent with observations in other mangrove ecosystems, where DOC concentrations increase with mangrove development. For example, in the mangroves of French Guiana, DOC concentrations ranged from approximately 8 mg L^−1^ in pioneer mangroves to about 300 mg L^−1^ in senescent mangroves (Marchand et al. 2006). This trend reflects a progressive increase in soil OM in both the solid and dissolved phases through forest development. These concentrations are higher than those found in Australian mangrove soils, with values ranging from 4 to 48 mg L^−1^, and which vary with the hydroperiod and stand position in the intertidal zone (Boto et al. 1989). Hydroperiod and porewater salinity strongly influence mangrove species distribution (Ball 1980; Duke 1992; Wang et al. 2024) but also organic carbon accumulation (Kristensen et al. 2017). However, in Ouvéa, no clear relationship was observed between solid and dissolved carbon concentrations (Online Resource 5) (Spearman’s correlation: − 0.28, *p*-value > 0.05) and salinity (Spearman’s correlation: − 0.21, *p*-value > 0.05). The semi-enclosed nature of the mangrove led to small tidal fluctuations (Online Resource 2) and relatively low porewater salinity, similar to that of the adjacent lagoon (35.5) (Bonvallot et al. 2013). This contrasts with other mangroves, such as those in Australia, where salinity levels can reach 90 (Ball 1998), or those in the Caribbean, where high porewater salinity (> 50) suggests evaporation of stagnant seawater (Medina-Calderón et al. 2021). The salinity observed in our study can be attributed to rapid water renewal within the mangrove, facilitated by the sandy, permeable soils of the karstic system, which promote efficient subsurface water circulation. This reduces water residence time and minimizes evaporation. This suggests a significant export of DOM from the mangrove to the creek and then to the lagoon of Ouvéa. This assumed rapid residence time would also explain the absence of vertical gradients.

Surprisingly, beneath mature *B. gymnorhiza* trees, despite very high TOC concentrations, with values of up to 30%, DOC concentrations were among the lowest of the studied sites (32 mg L^−1^). This contrasting accumulation between the solid and dissolved phases may result either from limited DOM production, rapid DOM decomposition, or vertical or lateral DOM export as soon as it is produced. TOC concentrations were also positively correlated with the HIX index (Spearman’s correlation: 0.64, *p*-value < 0.001) and compound C4 (Spearman’s correlation: 0.69, *p*-value < 0.001), both indicators of humified and mature organic matter, indicating an accumulation of aged material under mature *B. gymnorhiza* trees (Online Resource 5). The geomorphology of the mature *B. gymnorhiza* site may be responsible for the soil organic accumulation limiting the export. In fact, the mature *B. gymnorhiza* trees develop in a basin-like area. The higher water content measured at the mature *B. gymnorhiza* site compared to the other mangrove stands, resulting from TOC accumulation (Spearman’s correlation: 0.93, *p*-value < 0.001), may induce greater water retention within the soil, and thus limited export to the mangrove creek (Online Resource 5). This organic accumulation increases soil acidity, as evidenced by the lowest pH observed at this site (Spearman’s correlation: − 0.41, *p*-value < 0.05) (Online Resource 5). In mangrove soils, acidification can result from organic matter mineralization or sulphide oxidation (Clark et al. 1998). However, in this study, as the conditions are only mildly anoxic (Fig. 2), and despite the observation of sulphates (anhydrite) in the superficial part of the sampled core (unpublished data), their low content made sulphide oxidation unlikely to be a major factor. This increased acidity, influencing carbonate rocks dissolution, may contribute to the basin-like geomorphological feature observed at the mature *B. gymnorhiza* site. Additionally, the absence of sand grains (unpublished data) from the nearby channel further emphasizes the limited inputs and exports of matter from this mangrove stand. The most likely hypothesis for this site is reduced DOM production during diagenetic processes, conducting to an accumulation of older, more humified OM. Despite the absence of significant differences, an increase in DOC concentrations with depth can be observed (Online Resource 6). This is consistent with the quality of the organic matter observed and the significant contribution of humic compounds C4 and C2. The presence of arbuscular mycorrhizal fungi, conversely to the *R. apiculata*, may inhibit the development of other micro-organisms (Welc et al. 2010) and may result in a lower biological activity and thus DOC production.

**Fig. 9:**
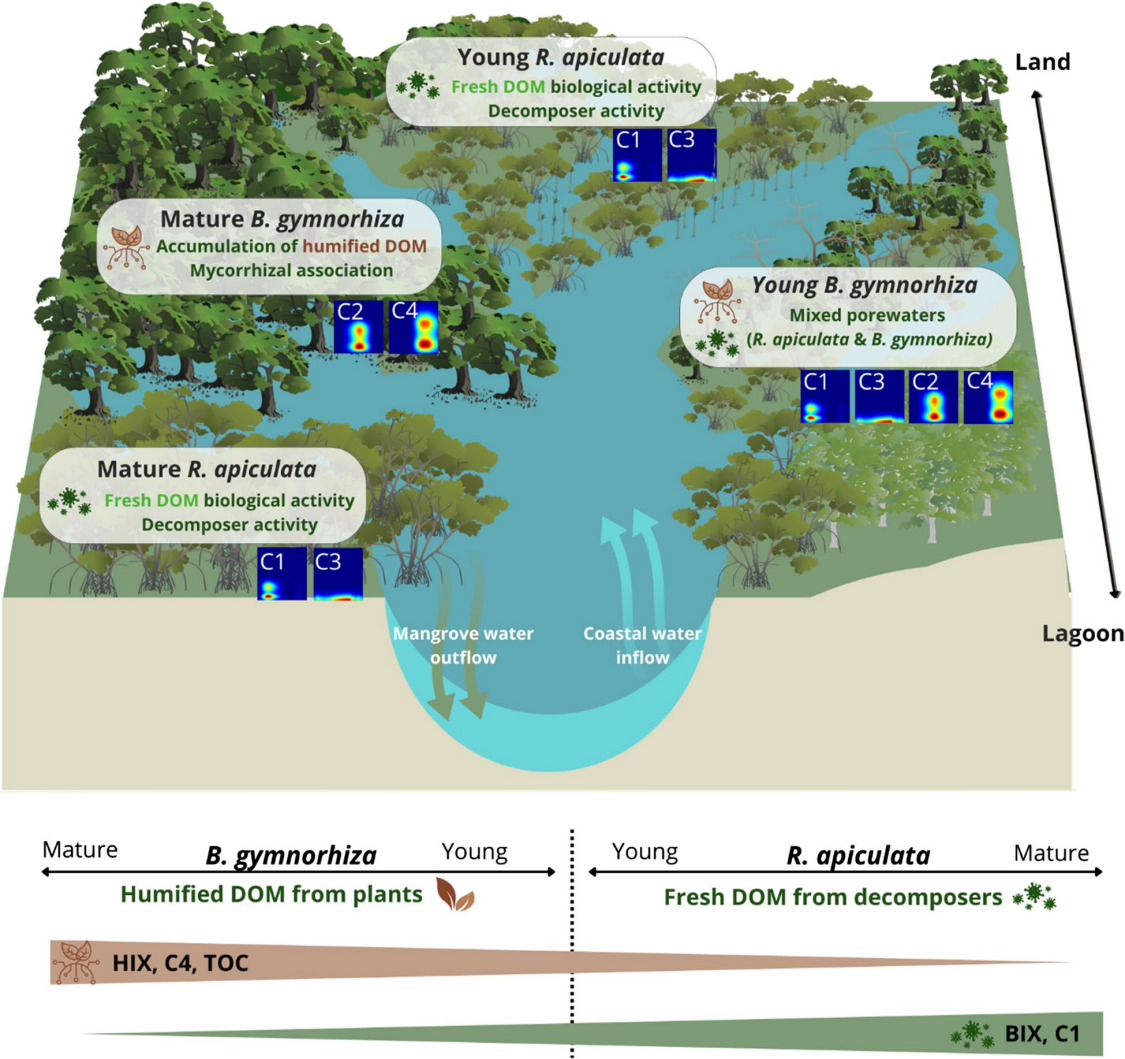
Conceptual representation of FDOM component distribution and associated biogeochemical parameters across four mangrove sites. Components C4 and C2 are primarily associated with mature *B. gymnorhiza*, while components C1 and C3 are common to both *R. apiculata* sites. The young *B. gymnorhiza* site exhibits a mix of FDOM components from both *B. gymnorhiza* (C4 and C2) and *R. apiculata* (C1 and C3), reflecting its transitional status as *B. gymnorhiza* trees are gradually replaced by young *R. apiculata*. The brown and green bands at the bottom represent trends in porewater quality and DOM chemistry along the gradient from mature *B. gymnorhiza* (left) to mature *R. apiculata* (right), the thicker the band, the larger the value. Inspired by Xiao et al. (2023) and Knoke et al. (2024). This figure was produced using Canva pro and the Integration and Application Network (IAN, ian.umces.edu/media-library)

To summarize findings, a conceptual representation (Fig. 9) of biogeochemical parameters and the FDOM sources along the Ouvéa mangrove, illustrates how the mangrove species can influence the DOM dynamics along the mangrove ecosystem. The differences in both quality and quantity of organic matter among mangrove species in this area could be attributed to the nature of the organic matter produced and the biological degradation processes that follow. Differences in OM sources such as the presence of decomposers under *R. apiculata* C1, *E*_2_/*E*_3_, *a*_442_; Table 1; Online Resource 3) or terrestrial material (C2, C4; Fig. 5) derived from vascular plants beneath *B. gymnorhiza* significantly influence the quality of DOM present in mangrove soils. These differences also extend to the quantity of OM, with a higher TOC concentration observed under mature *B. gymnorhiza*. However, this trend does not appear in dissolved forms (Fig. 3). While factors like the export of material through hydrodynamic currents or reduced diagenetic production were considered, the retained hypothesis is that the site’s basin-like geomorphology restricts exchanges. The differences in the nature and freshness of the material are also highlighted. Fresher material is observed under *R. apiculata* (as shown in Figs. 6 and 7b, BIX), while older material tends to accumulate under *B. gymnorhiza* (HIX; Fig. 7a). These variations could be attributed to biological differences associated with the tree species themselves. In fact, the ability to form associations with arbuscular mycorrhizal fungi may result in the increased production of fluorescent compounds such as C2 and C4 by the diagenetic products of tannin but may inhibit the development of other micro-organisms limiting biological activity and thus DOM production.

## Conclusion and perspectives

This study demonstrates the variability in terms of quality and quantity of DOM under different mangrove stands in a carbonate karstic context. Mangrove species appear to be the dominant factor in explaining these differences, rather than environmental factors such as salinity or acidity, which showed minimal variation across stands. Indeed, *R. apiculata* and *B. gymnorhiza* display distinct DOM profiles: *R. apiculata* stands are marked by higher levels of protein-like and fulvic-like fluorescence (C1 and C3), indicating fresher and more bioavailable OM. In contrast, *B. gymnorhiza*, particularly in mature stands, shows a higher presence of humic-like fluorescence (C4), associated with more humified and older OM. These findings suggest that DOM characteristics under each mangrove species are closely linked to differences in leaf litter composition specifically, and decomposition rates. *R. apiculata*, with a lower C:N ratio, supports faster OM turnover, while *B. gymnorhiza*, with its capacity for symbiotic relationships with arbuscular mycorrhizal fungi, sustains a more humified DOM pool as a result of reduced microbial activity. Thus, it is the species-specific properties of the mangroves themselves, rather than abiotic factors, that drive the quality and quantity of OM in these stands. Our study highlights the importance of tree stands (potential AMF associations and geomorphology) in determining DOM composition in mangrove soils, adding valuable insights into how species-specific traits shape biogeochemical processes in these unique ecosystems. Additionally, research could focus on measuring OM concentrations in rainwater and different wells across Ouvéa to assess groundwater quality in the karst system. Analysing groundwater through radon isotope studies could also help accurately determine residence times and quantify water dynamics in the mangrove ecosystem, as demonstrated in previous studies (Gleeson et al. 2013; Li et al. 2009; Maher et al. 2013; Martinez et al. 2015; Taillardat et al. 2018). Future studies incorporating molecular characterization techniques, along with tidal cycle monitoring, could further elucidate these relationships by capturing the influence of tidal dynamics on organic matter horizontal export and transformation. This approach would provide a deeper understanding of DOM fluxes and their ecological implications within and beyond mangrove ecosystems.

## Supplementary Information

Below is the link to the electronic supplementary material.

Supplementary file 1 (PDF 647 KB)

## Data Availability

The authors declare that the data supporting the findings of this study are available from the corresponding author upon reasonable request.
