# Supplementary material for: Variability of the optical signatures of dissolved organic matter in soils of different mangrove stands (Ouvéa, New Caledonia)
Source: Environ Sci Pollut Res Int. 2025 Apr 23;32(19):12086–107. doi: 10.1007/s11356-025-36373-9 (PMC12049311; doi:10.1007/s11356-025-36373-9)

Supplementary Material

Variability of the optical signatures of dissolved organic matter in soils of different mangrove stands (Ouvéa, New Caledonia)

Mouras Naïna[1,2], Lemonnier Hugues[2], Crossay Thomas[1], Gututauava Kapeliele[1], Mathian Maximilien[1], Robin Sarah Louise[1], Tardivel Océane[1], Marchand Cyril[1]

[1] Institut de Sciences Exactes et Appliquées (ISEA EA7484), Université de la Nouvelle-Calédonie, 145 Avenue James Cook, Nouville, BP R4 98851, Nouméa Cedex, New Caledonia

[2] Ifremer, UMR 9220 ENTROPIE (IRD, Univ. Réunion, IFREMER, Univ. Nouvelle-Calédonie, CNRS), Nouméa, New Caledonia / FRANCE

*Corresponding author: naina.mouras@ifremer.fr ; naina.mouras@unc.nc

Summited for Environmental Science and Pollution Research

Online Resource 1: Distribution of Clay, Silt, and Sand in the soils of the four Ouvéa mangrove sites.

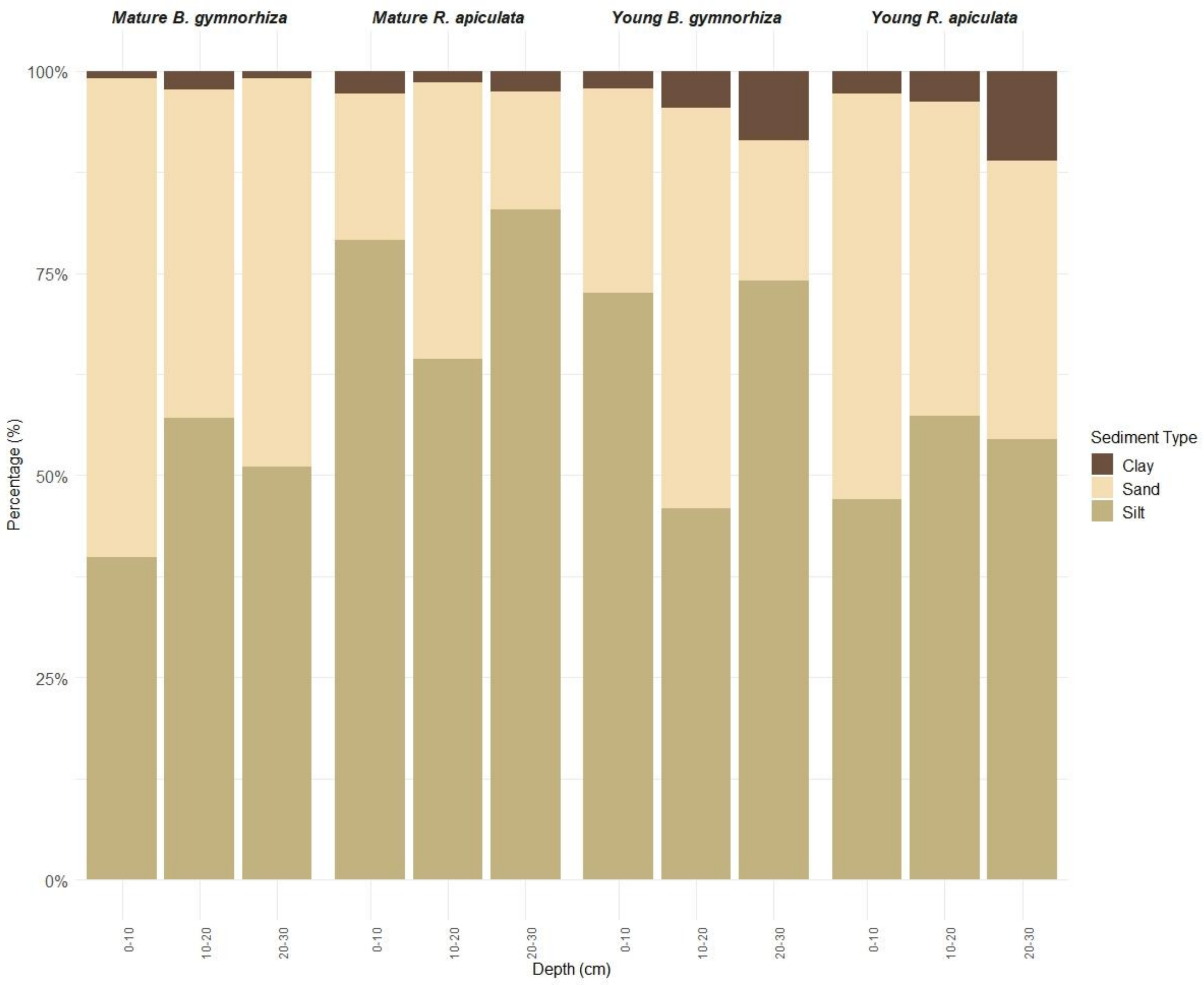

Online Resource 2: Tidal cycle in the semi-enclosed mangrove. A: The map with the localisation of sensor immerged 6 months between May to October 2023. B: the variation in cm of the elevation between the sensor outside the mangrove (St. Joseph) and inside the mangrove system. C: The tidal cycle during the TRACETS campaign May 2023 with water level in m for the two sensors

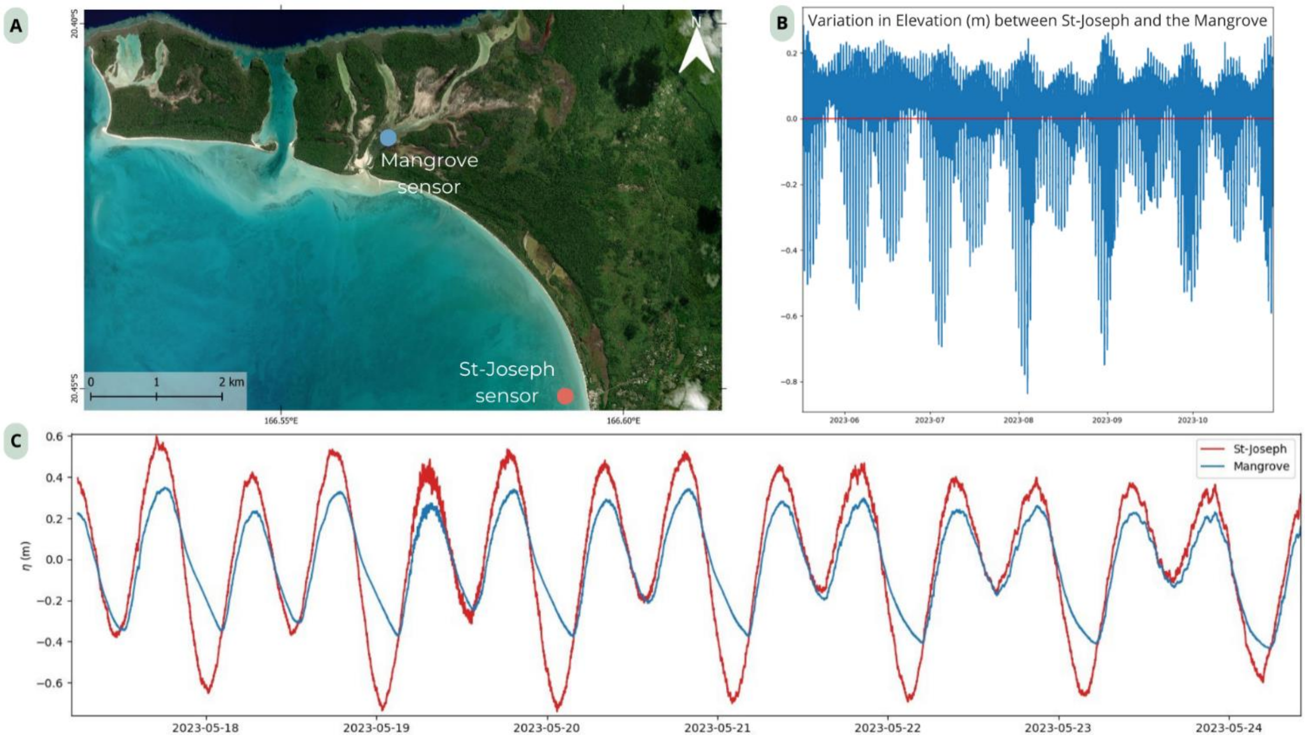

Online Resource 3: Distribution of FDOM components across the four mangrove stands (Mature and Young) for the two dominant species, B. gymnorhiza and R. apiculata, as a function of soil depth

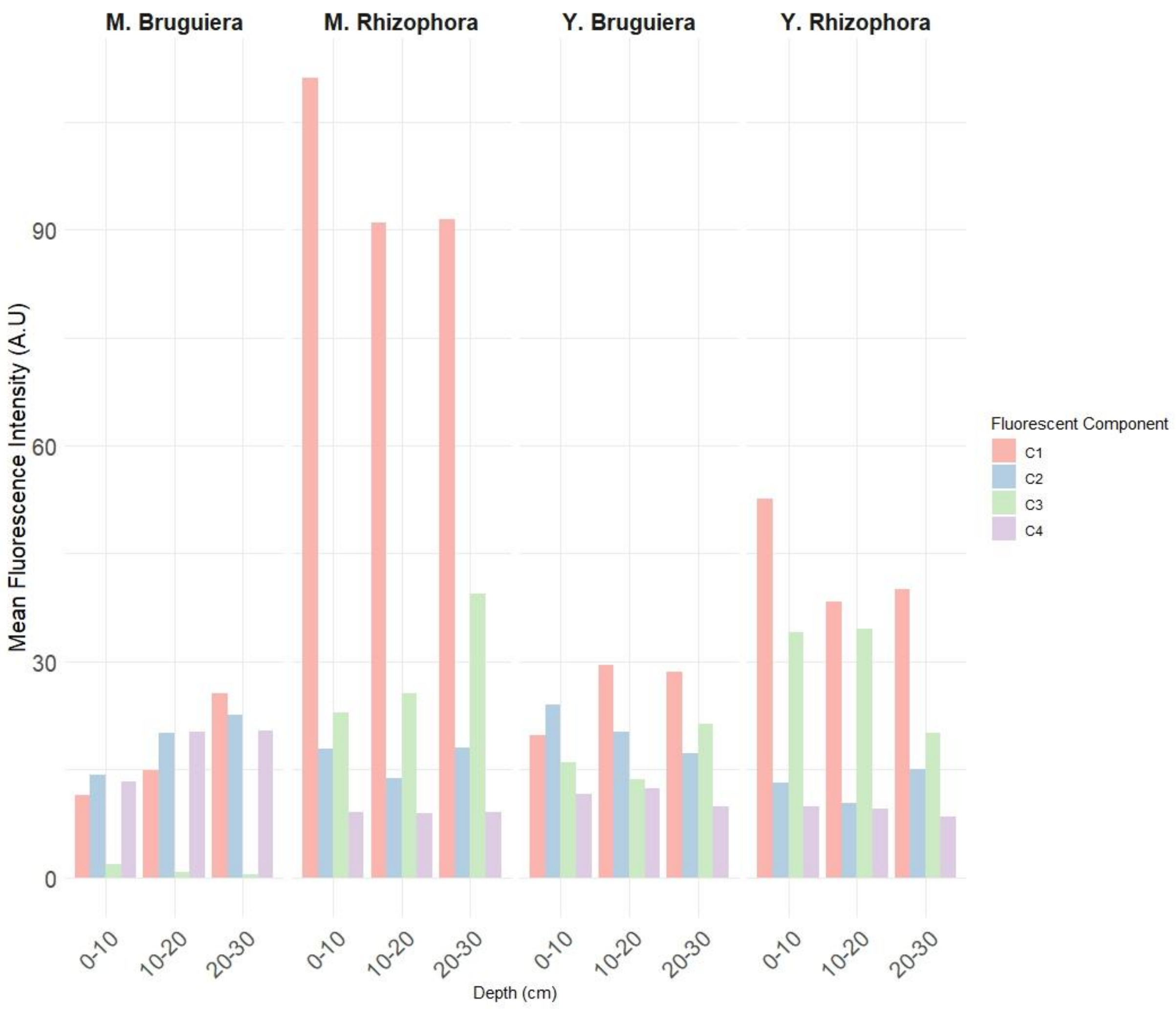

Online Resource 4: Boxplots representing the HIX (A) and BIX (B) indices calculated for the 4 sites (MR, MB, YR, YB) in function of the depth variation. The average value is indicated by a black diamond, and the median by a black bar. Note that the y-axes are not the same between the indices.

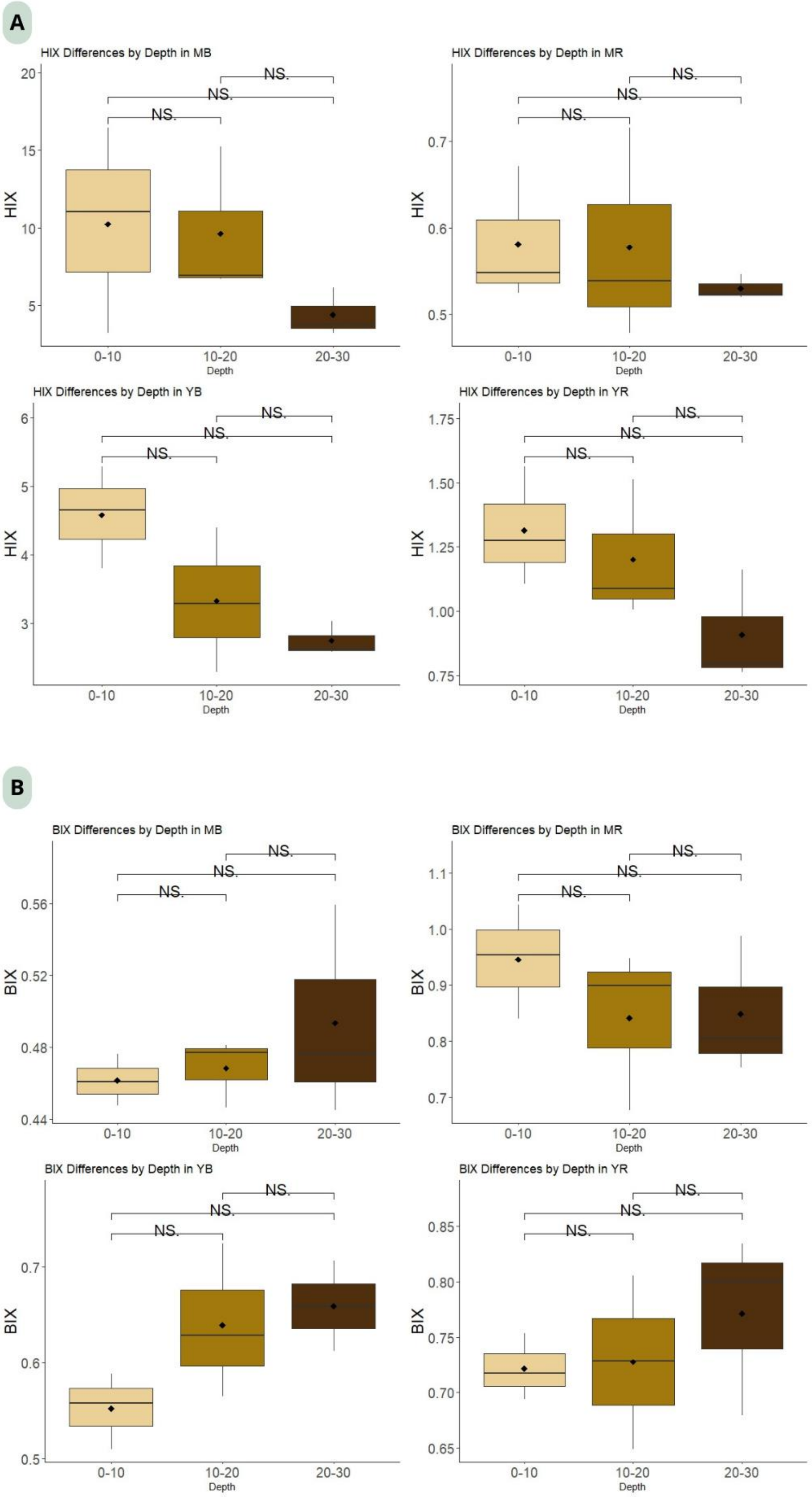

Online Resource 5: Spearman correlation matrix showing the relationships between all parameters, including DOM-related variables and environmental factors. The colours represent Pearson's correlation coefficients, ranging from red (negative correlations) to blue (positive correlations), with only statistically significant values (p < 0.05) displayed in the matrix.

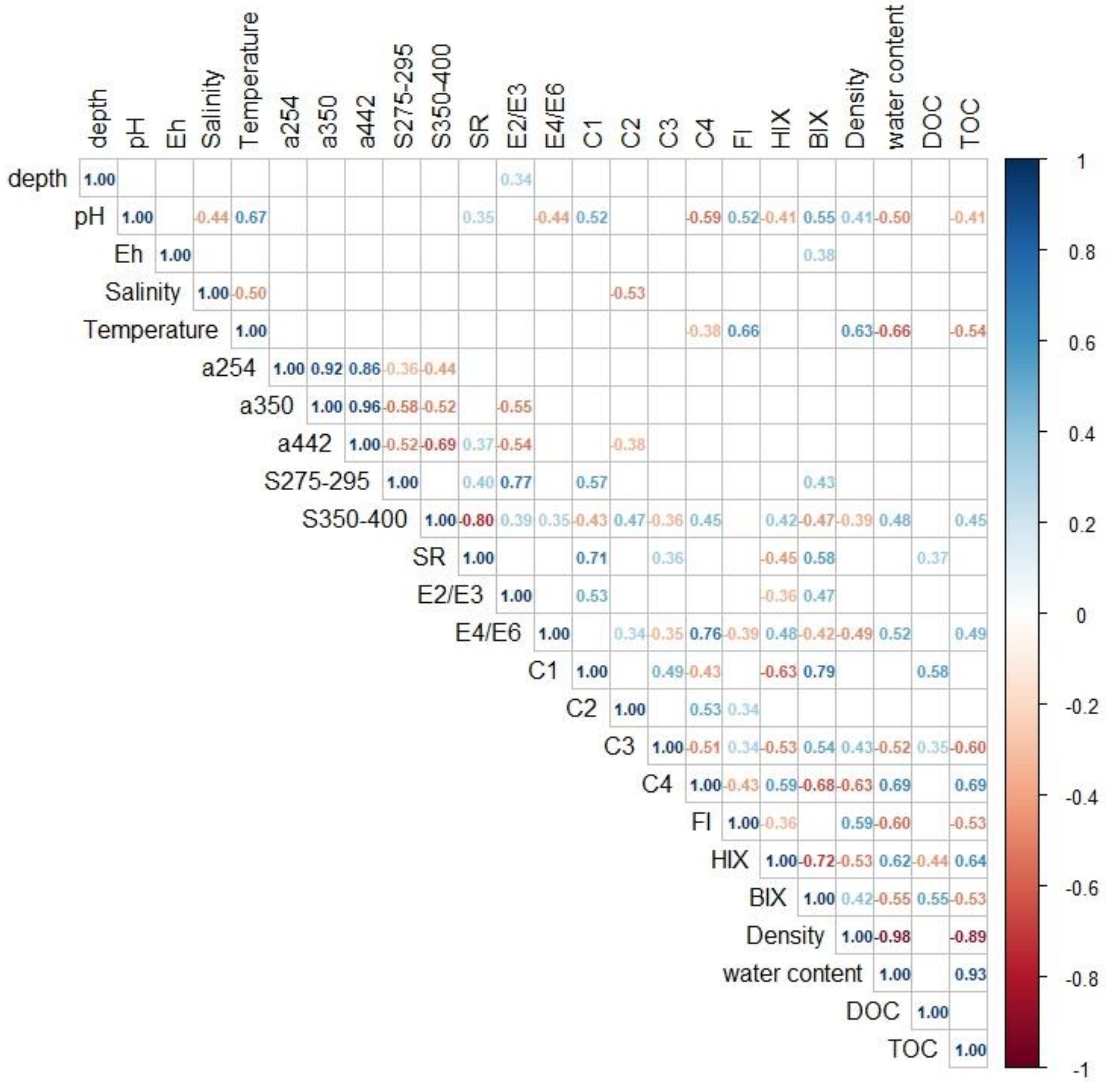

Online Resource 6: Boxplots representing the DOC (mg. L-1) for the 4 sites (MR, MB, YR, YB) in function of the depth variation. The average value is indicated by a black diamond, and the median by a black bar. Note that the y-axes are not the same between the indices.

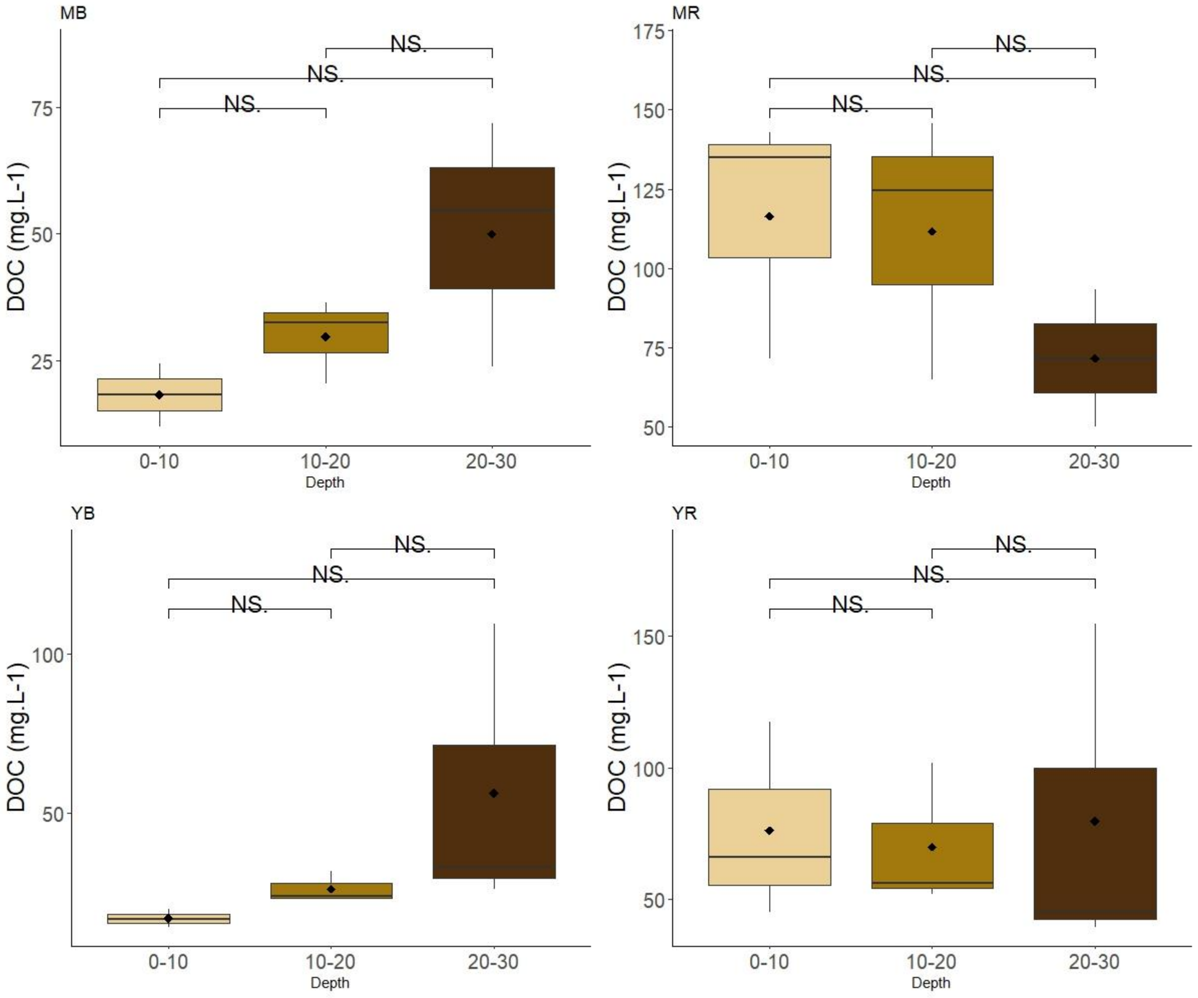

Supplement: Supplementary file 1 — (PDF 647 KB) [file 11356_2025_36373_MOESM1_ESM.pdf]
